# NOD2 and reproduction-associated NOD-like receptors have been lost during the evolution of pangolins

**DOI:** 10.1007/s00251-021-01230-9

**Published:** 2021-11-01

**Authors:** Margarita Salova, Wolfgang Sipos, Erwin Tschachler, Leopold Eckhart

**Affiliations:** 1 Department of Dermatology, Medical University of Vienna, Vienna, Austria; 2 Clinical Department for Farm Animals and Herd Management, University of Veterinary Medicine Vienna, Vienna, Austria

**Keywords:** NOD2, NLRP, Innate immunity, Inflammation, Pangolin, Zoonosis, Gene loss

## Abstract

NOD-like receptors (NLRs) are sensors of pathogen-associated molecular patterns with critical roles in the control of immune responses and programmed cell death. Recent studies have revealed inter-species differences in mammalian innate immune genes and a particular degeneration of nucleic acid sensing pathways in pangolins, which are currently investigated as potential hosts for zoonotic pathogens. Here, we used comparative genomics to determine which NLR genes are conserved or lost in pangolins and related mammals. We show that *NOD2*, which is implicated in sensing bacterial muramyl dipeptide and viral RNA, is a pseudogene in pangolins, but not in any other mammalian species investigated. *NLRC4* and *NAIP* are absent in pangolins and canine carnivorans, suggesting convergent loss of cytoplasmic sensing of bacterial flagellin in these taxa. Among NLR family pyrin domain containing proteins (NLRPs), skin barrier-related *NLRP10* has been lost in pangolins after the evolutionary divergence from Carnivora. Strikingly, pangolins lack all NLRPs associated with reproduction (germ cells and embryonic development) in other mammals, i.e., *NLRP2*, *4*, *5*, *7*, *8*, *9*, *11*, *13*, and *14*. Taken together, our study shows a massive degeneration of NLR genes in pangolins and suggests that these endangered mammals may have unique adaptations of innate immunity and reproductive cell biology.

**Supplementary information:**

The online version contains supplementary material available at 10.1007/s00251-021-01230-9.

## Introduction

**Fig. 1:**
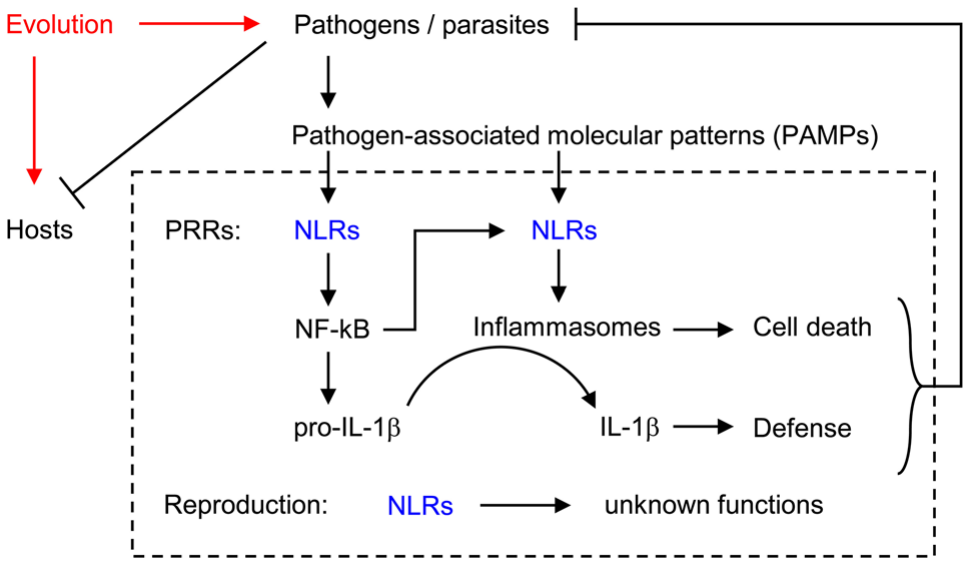
Roles of NOD-like receptors (NLRs). NLRs are involved in the activation of NF-kB-dependent gene expression and inflammasome-dependent cell death and immune responses. Besides their role in the defense against pathogens, NLRs have also unknown functions in the reproductive system. Evolution has shaped host–pathogen interactions and control mechanisms of reproduction in mammals

Nucleotide-binding oligomerization domain (NOD)-like receptors (NLRs) are a family of proteins involved in the defense against pathogens and in reproduction (Fig. 1). NLRs are characterized by the presence of a NOD, also known as NACHT domain, which is followed by leucine-rich repeats (LRRs). The NOD/NACHT domain mediates self-oligomerization upon binding of a ligand to LRRs. In addition, NLRs contain other domains such as 1 or 2 caspase recruitment domains (CARDs) or a pyrin domain (PYD), which mediate interactions with other proteins to control the initiation of inflammation, programmed cell death and other processes (Inohara et al. 2005; Fritz et al. 2006; Ting et al. 2008; Zhang et al. 2010; Elinav et al. 2011; Zhong et al. 2013; Geddes et al. 2009; Heim et al. 2019; Kienes et al. 2021; Danis et al. 2021). Regulators of immune defense have played critical roles in the evolution of host–pathogen interactions, leading to the diversification of defense strategies at the molecular level (Danilova 2006; Eckhart et al. 2005; Zhang et al. 2010; Chakraborty and Ghosh 2020).

The main subfamilies of NLRs are the NLR family CARD domain containing (NLRC) proteins and the NLR family pyrin domain containing (NLRP) proteins. Humans have 5 NLRCs (NOD1, NOD2, NLRC3-5) and 14 NLRPs (NLRP1-14). The primordial function of NLRs is the detection of pathogen-associated molecular patterns (PAMPs) and damage-associated molecular patterns (DAMPs), which include bacterial cell wall components, such as fragments of peptidoglycan that are sensed by NOD1 and NOD2 (Philpott et al. 2014; Wolf and Underhill 2018), viral RNAs which are sensed by NOD2 and NLRP6 (Sabbah et al. 2009; Wang 2015; Liu and Gack 2020), and others (Kuss-Duerkop et al. 2020; Pei et al. 2021). NLRs were originally identified as activators of inflammation and immune responses, but later research has demonstrated anti-inflammatory roles of several NLRs, such as NLRC3 (Li et al. 2019) and NLRP12 (Williams 2005; Chen et al. 2019).

Besides functions in innate immunity, NLRPs are implicated in germ cell biology and early embryonic development. There are nine human NLRPs that appear to have functions related to reproduction: *NLRP2*, *NLRP4*, *NLRP5*, *NLRP7*, *NLRP8*, *NLRP9*, *NLRP11*, *NLRP13*, and *NLRP14* (Tian et al. 2009; Zhang et al. 2008; Abe 2017; Amoushahi et al. 2019; Yin et al. 2020). These NLRP genes, which are phylogenetically distinct from other NLRPs (Tian et al. 2009), are expressed in germ cells and pre-implantation embryos (Zhang et al. 2008). Gene knockout studies showed that *NLRP2* controls age-associated maternal fertility (Kuchmiy et al. 2016), *NLRP5* is required for early embryonic development (Tong et al. 2000), and *NRLP14* is essential for differentiation of spermatogonial stem cells in mice (Yin et al. 2020). The mechanisms of action of reproduction-associated NLRPs are elusive.

Pangolins (order: Pholidota) are nocturnal mammals that feed on ants and termites. Phylogenetically, they are most closely related to carnivorans (order: Carnivora), a clade comprised of cat-like (Feliformia) and dog-like (Caniformia) mammals with the latter including canines, bears, procyonids (raccoons and relatives), mustelids (weasels and relatives), skunks (mephitids), red pandas (ailurids), and pinnipeds. The body of pangolins is covered by keratinous scales which serve as a protective armor (Choo et al. 2016; Li et al. 2020). Few comparative studies of the mammalian immune defense have included pangolins, but the interest in pangolins has increased recently due their possible role as intermediate hosts for the pandemic severe acute respiratory syndrome coronavirus 2 (SARS-CoV-2), a betacoronavirus with a single-stranded RNA genome (Lam et al. 2020; Xiao et al. 2020; Zhang et al. 2020; Andersen et al. 2020). Recently, we have reported that interferon-induced with helicase C domain 1 (IFIH1)/MDA5, Z-DNA-binding protein (ZBP1), cyclic GMP-AMP synthase (cGAS), and stimulator of interferon genes (STING), which initiate innate immune responses to intracellular nucleic acids, have been lost during the evolution of pangolins (Fischer et al. 2020a, b). Likewise, toll-like receptor (TLR) 5, an endosomal receptor of bacterial flagellin, is absent in pangolins (Sharma et al. 2020).

Here we investigated whether NLR genes are conserved in pangolins and found that *NOD2* and several other NLRs have underdone pseudogenization or were entirely lost, indicating that immune responses to specific pathogens and NLR-dependent processes in the reproduction system are altered in pangolins.

## Materials and methods

Genes were identified in the genome sequences of the Malayan pangolin (*Manis javanica*), Assembly: ManJav1.0 (GCA_001685135.1), submitted by the International Pangolin Research Consortium (Choo et al. 2016); Chinese pangolin (*M. pentadactyla*), Assembly: M_pentadactyla-1.1.1 (GCA_000738955.1), submitted by Washington University; and tree pangolin (*Phataginus tricuspis*, previously named *Manis tricuspis*), Assembly: ManTri_v1_BIUU (GCA_004765945.1), submitted by Broad Institute. At the time of this study (July 2021), GenBank gene annotations were available for *M. javanica* (NCBI Manis javanica Annotation Release 100) and *M. pentadactyla* (NCBI Manis pentadactyla Annotation Release 100) but not for the other species of pangolins. Other nucleotide sequences were downloaded from GenBank, and accession numbers are indicated in the text.

Sequence similarities were identified with the Basic Local Alignment Search Tool (BLAST) (Altschul et al. 1990). Nucleotide sequences were translated into amino acid sequences using the Translate tool at the Expasy website of the Swiss Institute of Bioinformatics (https://web.expasy.org/translate/). Sequences were aligned with MUSCLE (https://www.ebi.ac.uk/Tools/msa/muscle/) and Multalin (http://multalin.toulouse.inra.fr/multalin/). Phylogenetic relationships and divergence times were obtained from the Timetree website (www.timetree.org) (Hedges et al. 2015).

## Results

### NOD2 is inactivated by gene mutations in pangolins

**Fig. 2:**
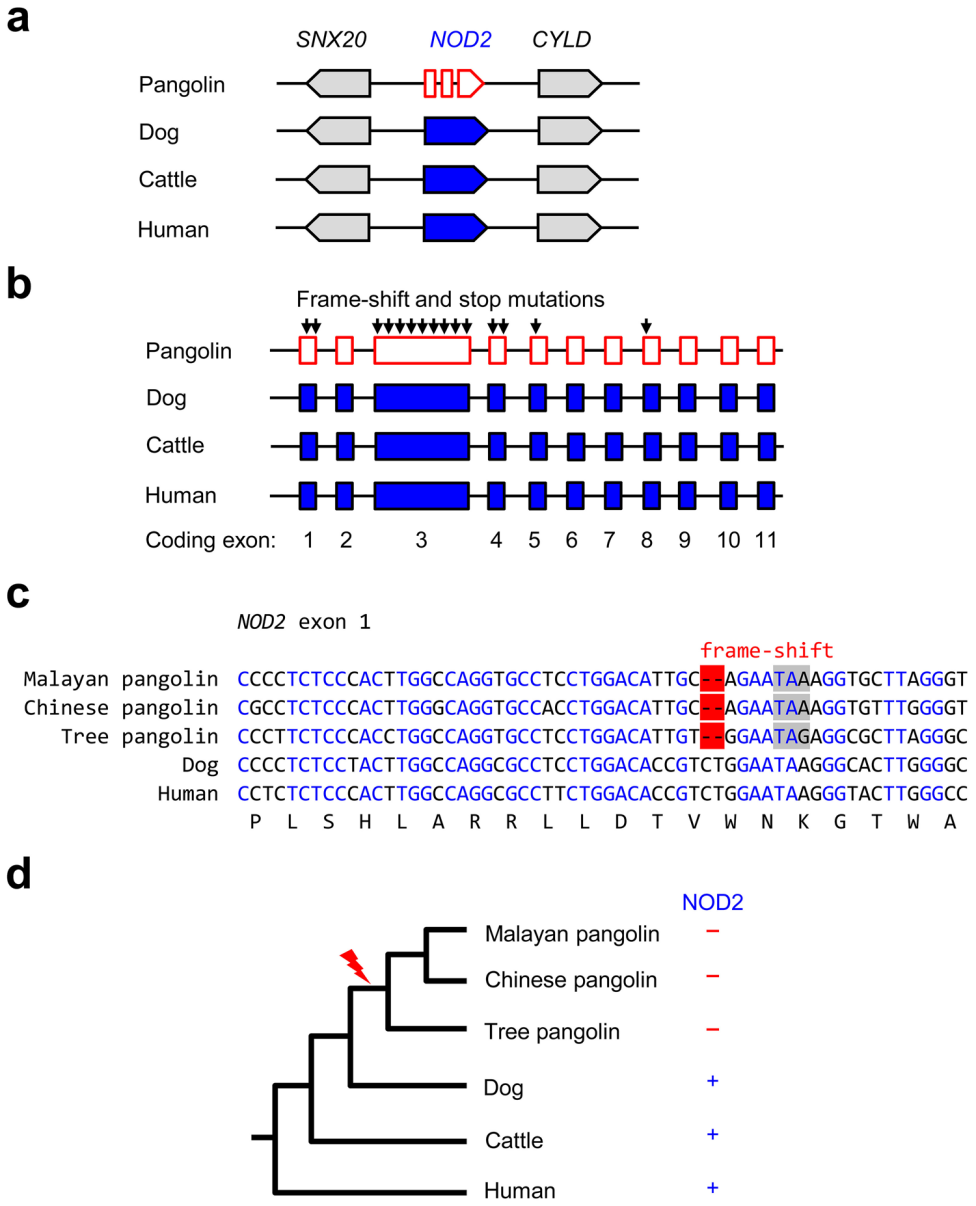
*NOD2* is a pseudogene in pangolins. **a** Gene locus of *NOD2* in the Malayan pangolin, dog, cattle, and human. Genes are represented by arrows pointing into the direction of transcription. The mutated NOD2 gene is shown as a broken arrow. **b** Inactivating mutations in multiple exons of pangolin *NOD2*. Positions of frame-shift and premature stop mutations are indicated by vertical arrows. Exons of *NOD2* of pangolin, dog, cattle and human are shown as boxes. **c** Nucleotide sequence alignment of a segment of exon 1 that contains a conserved frame-shift mutation (red shading) leading to a premature stop codon (grey shading) in three species of pangolins. Nucleotides identical in all species are shown with blue fonts. **d** The absence ( −) or presence ( +) of intact *NOD2* in 6 species was used to map the *NOD2* gene loss (flash symbol) on a simplified phylogenetic tree of mammals. Species: Malayan pangolin (*Manis javanica*), Chinese pangolin (*Manis pentadactyla*), tree pangolin (*Phataginus tricuspis*), dog (*Canis lupus familiaris*), cattle (*Bos taurus*), and human (*Homo sapiens*)

We investigated foreign nucleic acid response genes in the Malayan pangolin, a species that is considered a potential intermediate host of the SARS-CoV-2 (Lam et al. 2020; Xiao et al. 2020; Zhang et al. 2020; Niu et al. 2021). Comparative analysis of *NOD2*, which had been reported to be involved in sensing single-stranded RNA (Sabbah et al. 2009), showed that a *NOD2* gene locus is present in pangolins, dog, cattle, and human (Fig. 2a). However, frame-shift and premature stop mutations were detected in exons 1, 3, 4, 5, and 8 of *M. javanica NOD2* (Fig. 2b). Analysis of genome sequences of two other pangolin species, the Chinese and the tree pangolin, revealed that inactivating mutations were also present in those species, whereby a frame-shift mutation leading to a premature stop of the reading frame in exon 1 was identified in all three species of pangolins investigated (Fig. 2c). By contrast, *NOD2* is intact in all other mammalian species investigated (Suppl. Fig. S1). These data suggest that *NOD2* has been inactivated by a gene mutation in the last common ancestor of pangolins (Fig. 2d).

### NLRC4 and NAIP are inactivated by gene mutations in pangolins

**Fig. 3:**
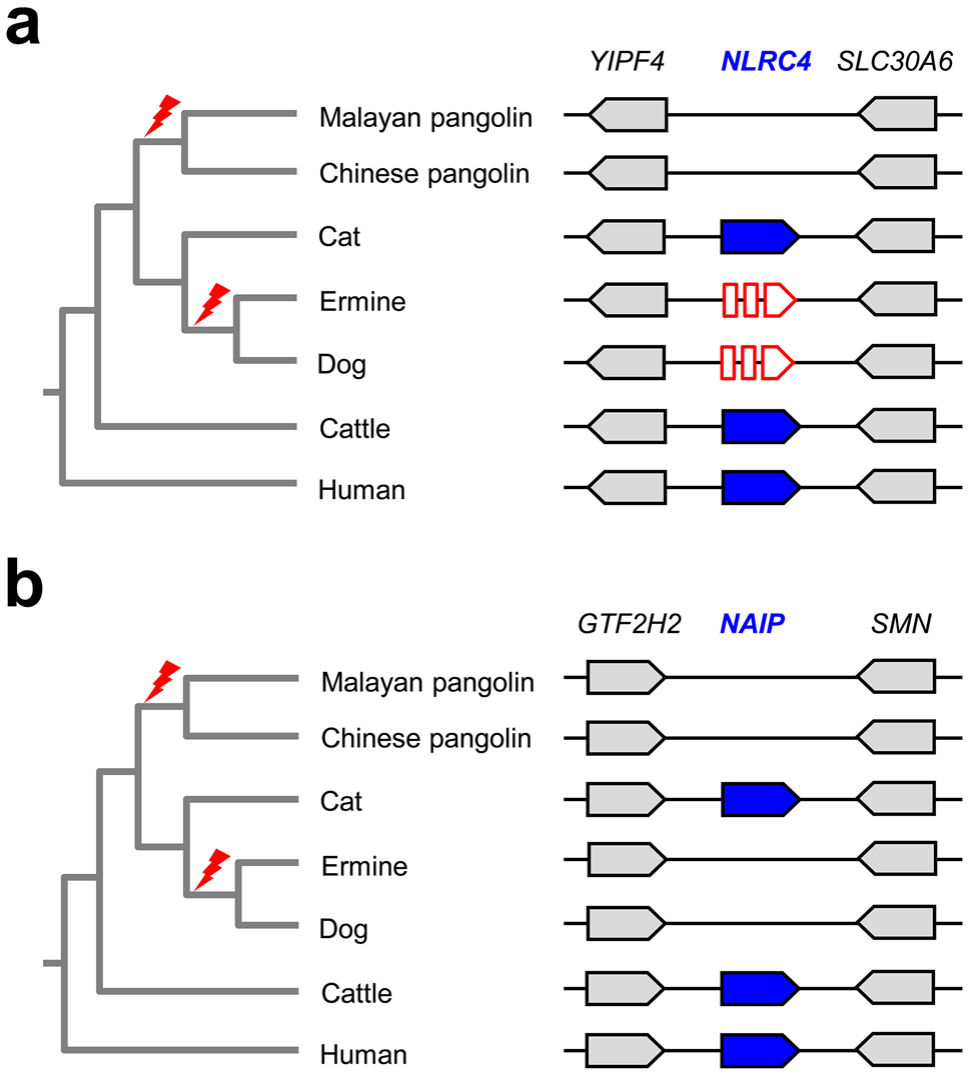
Absence of *NLRC4* and *NAIP* in pangolins. **a** Gene locus of *NLRC4*. Genes are represented by arrows pointing into the direction of transcription. Intact *NLRC4* genes are shown as blue arrows. Red broken arrows indicate inactivating mutations of *NLRC4* in caniformia, represented by dog and ermine. *NLRC4* is absent in pangolins. Loss of *NAIP* was mapped (red flash symbols) onto a simplified phylogenetic tree of mammals. **b** Gene locus of *NAIP*. Genes are represented by arrows pointing into the direction of transcription. Intact *NAIP* genes are shown as blue arrows. *NAIP* is absent in pangolins, dog, and ermine. Loss of *NAIP* was mapped (red flash symbols) onto a simplified phylogenetic tree of mammals. Species: Malayan pangolin (*Manis javanica*), Chinese pangolin (*Manis pentadactyla*), cat (*Felis catus*), ermine (*Mustela erminea*), dog (*Canis lupus familiaris*), cattle (*Bos taurus*), and human (*Homo sapiens*)

Next we investigated whether the inactivation of *NOD2* is unique among NLR family genes in pangolins. We analyzed the loci of NLR genes and flanking genes in human, cattle, dog, and the Malayan pangolin. The nucleotide sequences of all NLR gene homologs were screened for the presence of mutations that would disrupt the coding sequences. *NOD1*, *CIITA*, *NLRX1*, *NLRC3*, and *NLRC5*, which is comprised of 47 exons in the pangolin and encodes the longest of all NLR proteins with 1859 amino acids, are free from inactivating mutations in the four aforementioned species. By contrast, *NLRC4* was entirely absent from the genomes of pangolins (*M. javanica*, *M. pentadactyla*, *Phataginus tricuspis*) although both genes flanking *NLRC4* in the human genome are conserved (Fig. 3a). In line with the results of a previous study (Eckhart et al. 2009), *NLRC4* contains inactivating mutations in the dog. *NLRC4* is also a pseudogene in the ermine (*Mustela erminea*) but not in the cat. These data indicated that *NLRC4* was inactivated in the last common ancestor of pangolin and, by parallel evolution, in the last common ancestor of Caniformia (“dog-like” carnivorans, including dogs, mustelids, bears, pinnipeds, and others) (Fig. 3a).

Strikingly, the second receptor of intracellular flagellin, i.e., NLR family apoptosis inhibitory protein (NAIP), is also absent from pangolins and caniforms (Fig. 3b). We conclude that parallel evolution has led to the loss of both NLR proteins involved in intracellular flagellin detection in pangolins and caniforms.

### The majority of NLRP genes have been inactivated in pangolins

**Fig. 4:**
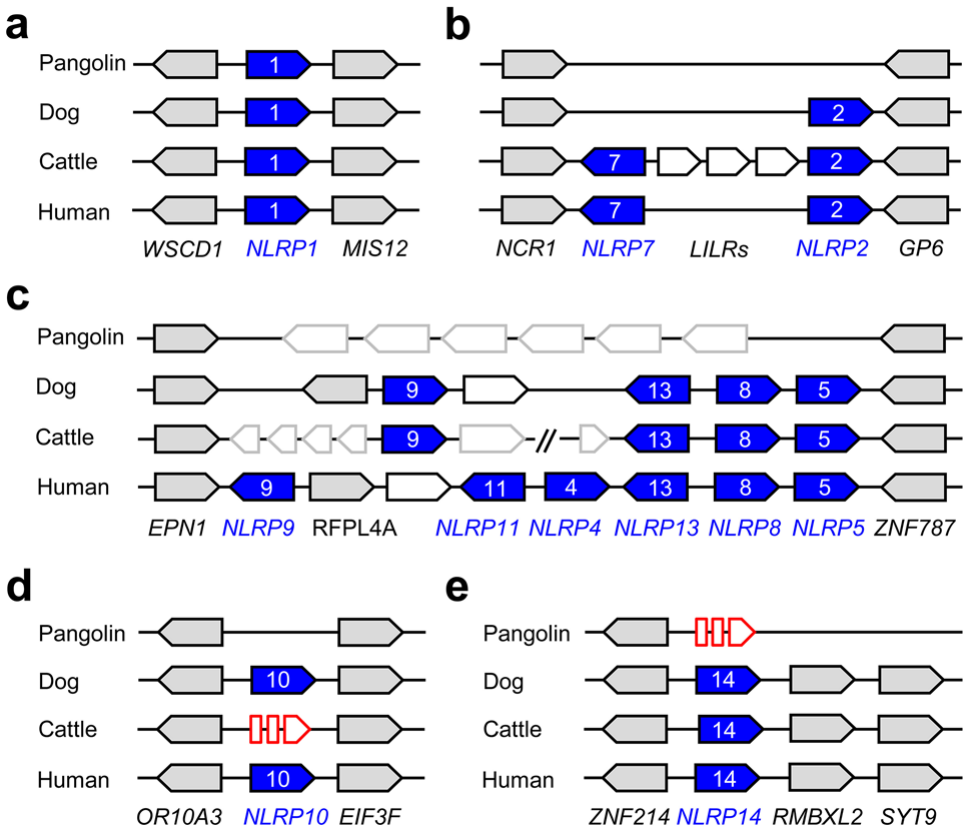
Absence of multiple *NLRP* genes in pangolins. Gene loci of *NLRP1* **a**, *NLRP2* and *NLRP7* **b**, *NLRP4*, *NLRP5*, *NLRP8*, *NLRP9*, *NLRP11*, and *NLRP13* **c**, *NLRP10* **d**, and *NLRP14* **e** of Malayan pangolin, dog, cattle, and human are schematically depicted. Intact NLRP genes are shown as blue arrows with white numbers indicating the number of the NLRP gene. Red broken arrows indicate NLRP genes that are inactivated by mutations. Grey arrows indicate evolutionarily conserved genes flanking NLRP genes. White arrows represent genes that are not conserved across species. Species: Pangolin (*Manis javanica*), dog (*Canis lupus familiaris*), cattle (*Bos taurus*), and human (*Homo sapiens*)

NLRP genes represent the largest group of NLR genes with 14 members in the human genome. Comparative genomics showed that *NRLP1* (Fig. 4a), *NLRP3*, *NLRP6*, and *NLRP12* are conserved in pangolins, whereas *NLRP2* and *7*, which are neighbors in the human genome (Fig. 4b); *NLRP4*, *5*, *8*, *9*, *11*, and *13*, which are clustered in the human genome (Fig. 4c); and *NLRP10* (Fig. 4d) are absent from the genomes of pangolins. Absence was confirmed by BLAST searches against entire genome sequences and against the genome region between orthologs of genes that flank the aforementioned NLRP genes in other mammalian species (Fig. 4b–d). An ortholog of *NLRP14* is present in pangolins, but its coding sequence is disrupted by inactivating mutations (Fig. 4e; Suppl. Fig. S2).

In total, there are only 8 intact NLR genes in the Malayan pangolin, as compared to 20 in human and 15 in both cattle and dog (Table 1). Remarkably, nine NLRPs that are predominantly or exclusively expressed in germ cells and early stages of embryonic development, i.e., *NLRP2*, *NLRP4*, *NLRP5*, *NLRP7*, *NLRP8*, *NLRP9*, *NLRP11*, *NLRP13*, and *NLRP14*, lack orthologs in pangolins (Table 1).

**Table 1:** Presence and absence of NLR genes in pangolins and other mammals

Gene	Ligand, function^1^	Main expression site (adult)	Human	Cattle	Dog	Pangolin	Gene evolution
NOD1	Ligand: DAP (peptidoglycan)	Lung and most other organs	+	+	+	+	Conserved
NOD2	Ligand: MDP (peptidoglycan)	Bone marrow, skin	+	+	+	−	Loss in pangolins
NLRC3	Ligand: DNA; inhibition	Lymph node, spleen	+	+	+	+	Conserved
NLRC4	Ligand: flagellin	Appendix, bone marrow	+	+	−	−	Loss in pangolins and Caniformia
NLRC5	MHC class I gene expression	Spleen, lymph node	+	+	+	+	Conserved
NLRP1	Proteolysis, dsRNA	Skin, spleen, lymph node	+	+	+	+	Conserved
NLRP2	Early embronic development	Placenta, testis, bladder	+	+	+	−	Loss in pangolins
NLRP3	Stimulus: Potassium efflux	Bone marrow	+	+	+	+	Conserved
NLRP4	Early embronic development	Testis	+	−	−	−	Origin in Euarchontoglires
NLRP5	Early embronic development	Testis	+	+	+	−	Loss in pangolins
NLRP6	Antimicrobial defense	Intestine, duodenum	+	+	+	+	Conserved
NLRP7	Early embronic development	Testis	+	+	−	−	Loss in pangolins and dog
NLRP8	Early embronic development	Testis, prostate	+	+	+	−	Loss in pangolins
NLRP9	Ligand: dsRNA	Prostate, testis	+	+	+	−	Loss in pangolins
NLRP10	Anti-inflammatory	Skin	+	−	+	−	Loss in pangolins
NLRP11	Anti-inflammatory	Testis	+	−	−	−	Origin in primates
NLRP12	Anti-inflammatory	Bone marrow	+	+	+	+	Conserved
NLRP13	Unknown	Testis	+	+	+	−	Loss in pangolins
NLRP14	Early embronic development	Testis	+	+	+	−	Loss in pangolins
NLRX1	Anti-inflammatory	Esophagus, skin	+	+	+	+	Conserved
CIITA	MHC class II gene expression	Bone marrow	+	+	+	+	Conserved
NAIP	Ligand: flagellin	Appendix, spleen	+	+	−	−	Loss in pangolins and Caniformia
		Number of NLRs:	20	15	15	8	
		Number of NLRPs:	14	11	11	4	

*DAP* γ-D-glutamyl-meso-diaminopimelic acid, *MDP* muramyl dipeptide, *ds* double-stranded

^1^Ligands and functions of several NLRPs are uncertain. Only subsets of proposed functions are listed

## Discussion

The results of this study show that pangolins lack numerous NLRs, suggesting that the evolution of pangolins was compatible with or even supported by the loss of these pattern recognition receptors and the associated defense processes. This study was focused on pangolins because (1) previous investigations had suggested a partial degeneration of antiviral and antimicrobial defense in these peculiar mammals (Kotze et al. 2016; Fischer et al. 2020a, b; Sharma et al. 2020) and (2) a better understanding of pathogen-host interactions in pangolins might help to evaluate the potential role of pangolins in the origin of zoonoses such as Covid-19 (Lam et al. 2020; Xiao et al. 2020; Zhang et al. 2020; Niu et al. 2021). Our findings lend support to the notion that the innate immune system of pangolins is unique among mammals and provide a basis for further investigations into the possible role of pangolins as reservoirs of pathogens that might benefit from the lack of NLR-dependent defense mechanisms.

*NOD2* is inactivated by multiple mutations in pangolins, whereas it is conserved in all other mammalian species investigated (Suppl. Fig. S1) and human *NOD2* loss-of-function mutations cause Crohn’s disease, a chronic inflammatory intestinal disease (Nayar et al. 2021). In non-mammalian vertebrates, *NOD2* is evolutionarily conserved in fish (Nayar et al. 2021) but not in reptiles (Choo et al. 2019). NOD2 is primarily required for antibacterial defense but has also been implicated in antiviral defense and general sensing of perturbations of cellular homeostasis, in particular the formation of sphingosine-1-phosphate (Pei et al. 2021). Recently, a drug that targets NOD2 was shown to have antiviral activity against SARS-CoV-2 and other RNA viruses (Limonta et al. 2021). It is conceivable that NOD2-dependent responses to specific intracellular bacteria or viruses are not required or even detrimental for pangolins, possibly due to unfavorable reactions against commensal microorganisms. Furthermore, NOD2-independent defense mechanisms may have evolved in pangolins. Studies on tissues or cells of pangolins are required to test these hypotheses.

Both cytosolic sensors of bacterial flagellin, i.e., NLRC4 and NAIP, have been lost in pangolins, making them in this regard similar to species of the clade Caniformia (dog-like carnivorans) (Eckhart et al. 2009) and pigs (Dawson et al. 2017; Sakuma et al. 2017). Strikingly, the endosomal receptor of flagellin, TLR5, has also been lost in pangolins (Sharma et al. 2020), suggesting that the response of pangolins to flagellated bacteria is uniquely degenerated. It is conceivable that these gene losses are linked to the evolution of a special gut microbiome due to the solely insectivorous diet or to the evolution of tolerance to specific pathogen groups in pangolins. Thus, it remains to be elucidated whether the response to flagellated bacteria, such as *Salmonella typhimurium*, *Legionella pneumophila*, and *Shigella flexneri*, with pathogenic potential in other species is suppressed in pangolins.

The number of NLRP genes varies among mammalian species (Tian et al. 2009). Until recently, the limited availability and quality of genome sequences has hampered a comprehensive study of NLRP genes across mammals. Our data show that pangolins have fewer NLRPs than dog, cattle, and humans. *NLRP4*, *NLRP11*, and possibly also *NLRP7* have originated after the divergence of the phylogenetic lineages leading to humans and pangolins, but the presence of *NLRP2*, *NLRP10*, and *NLRP14* in a common ancestor of humans and pangolins can be inferred from their distribution in other mammals (Table 1). Therefore, at least *NLRP2*, *NLRP10*, and *NLRP14* have been lost in pangolins. *NLRP10* is expressed predominantly in the skin where it is transcriptionally upregulated during epidermal cornification (Lachner et al. 2017). Keratinocyte cell death by cornification, like apoptosis and unlike pyroptosis, does not induce pro-inflammatory signaling, and we have put forward the hypothesis that *NLRP10* contributes to the suppression of inflammation during cornification (Eckhart and Tschachler 2018). *NLRP10* may have been lost in the course of the evolutionary adaptation of the integument in pangolins, which is characterized by an almost complete replacement of hairy skin by keratinous scales.

Remarkably, all nine reproduction-associated NLRPs of humans, i.e., *NLRP2*, *NLRP4*, *NLRP5*, *NLRP7*, *NLRP8*, *NLRP9*, *NLRP11*, *NLRP13*, and *NLRP14* (Tian et al. 2009), lack orthologs in pangolins. The loss of NLRPs that are associated with germ cell biology and embryonic development (Table 1) suggests that the reproduction of pangolins does not depend on NLRP-mediated processes, which are not understood at present but may include the control of inflammation (Amoushahi et al. 2019; Yin et al. 2020; Carriere et al. 2021). Deepening the knowledge on the physiology of reproduction and embryology of pangolins would be beneficial for conservation aspects of these highly endangered species as breeding efforts in zoos are scarce (Yang et al. 2007; Sipos and Lutonsky 2021).

In summary, the repertoire of NLRs is greatly reduced in pangolins as compared to other mammals, which indicates diversification of immune defense and reproduction-related processes during the evolution of different mammalian lineages. Pangolins, presumably anergic to a series of otherwise pathogenic agents, may carry distinct microbes and viruses that can be transmitted to other species and potentially give rise to as-yet-unknown zoonoses. Therefore, comparative studies of innate immunity in pangolins and other mammals are warranted.

## Supplementary information

Below is the link to the electronic supplementary material.

Supplementary file1 (PDF 801 KB)

## Data Availability

All data and material of this study are publicly available.
