# Supplementary material for: NOD2 and reproduction-associated NOD-like receptors have been lost during the evolution of pangolins
Source: Immunogenetics. 2021 Nov 1;74(2):261–8. doi: 10.1007/s00251-021-01230-9 (PMC8560141; doi:10.1007/s00251-021-01230-9)
Supplement: Supplementary file 1 — Supplementary file1 (PDF 801 KB) [file 251_2021_1230_MOESM1_ESM.pdf]

a

```
>XP_038610111.1 nucleotide-binding oligomerization domain-containing protein 2 [Tachyglossus
aculeatus]
MLPPESFWAQRSQLVTQLGRGSAETFESVLDFLLSWDVLTWEDYESVSIRGHPLSQLARRLLDIVGNKGGQSGELLVSALREAEREVRQAGGSRERLPEA
SGPIWDLQRQRPAIVRKIYGHLDDLMGLLIDRGFVSKYECDEIRLPIFTNSQQARRLLDLANAKENGMVEFLLHCVQQLPDLSAPLPDDGVFKKYQSKLR
ATLSAQSRFLSTYDGMENLCLEDVYTENVLEIREIDGASDRQRGPAPLGLPDLFDPGGCLNEDADTVLLVGGAGSGKTTFLQRLVWLWATGRAFQDCLLV
FPFSCRQLRCMRKPVSVKTLLFEHCCWADGRQRDIFQFVLDHPQRVLLTFDGFDEFKFKFAEGARHCSPTEPTSVQNLLVNLIQGNLLKGSRKVLSSRPE
AVTACLRKYIRKEVGLRGFSQEGIERFMRKHHGDPGVADQIVRLVRATPALRGLCHVPVFSWIVSRCHVELLQRWQGSSGGSLKTMTDMYFLIVRHLVLR
SPLEGNRGSAEGVWRGRLPALLRLGELALGGLGAGCYVFSDGQLQAVGVSTEDLSLGFLVPSKSSAGAAPQAHFEFLHVTFQCFLAALYLLLHSDVTPAA
VGHLFHRPRKCRVLDCLYPQLCIPAARREQGRPGPPLWGMETLDVQLTASFLAGLLSGANFSPLVEGCGLEALLRKRASARKCLAQGIKRHFGSIPPAVP
GELKSLHALPAFLWLIKSLYEMQDEGLARRALRGFKVEHVKLTYCGVGPAECAALAFVLRFLRHPVSLQLDYNSVGDVGLEQLLPCLKVCRALYLRDNNI
SDHGICKLVDQALLCDSFQKLALFHNKLTDDSAHSLAKLLKYKENFLALRLGNNHITAVGAKVLAEGLERNHSLQFLGLWGNAVGDEGAQALADALHGHR
SLKWLSLVGNDIGSVGAQALALMLGKNVVLEELCLEENRFSDQDVCSLAEGLKKNSTLKVLKLSNNNITCQGVVSLLQTLKKNDTLKSIWLRGNTFTLEE
IESLSYMDPRLLL

>XP_001519938.2 nucleotide-binding oligomerization domain-containing protein 2 isoform X1
[Ornithorhynchus anatinus]
MRRTMLPPETFRAQRSQLVAQLGRGSTETFESVLDFLLSWDVLTWEDYESVSVRGHPPSQLARRLLDIVGNKGGRSGELLVAAVREAEREVRRAGGSWER
LPEASGPIWDLQRQRPAVVRKIYGHLDDLLRLLLDRGFVSKYECDEIRLPIFTTSQQARRLLDLAKAKENGMVEFLLHRVQQLPDLPAPLPDDGVFEKYQ
SKLRATLSAQSRFLSTYDGMENLCLEDVYTENALEIRKIDGASEPWRKGPAPLGLPDLFDPGGSLNEDADTVLLVGGAGSGKTTLLQRLVWLWATGRAFQ
DCLLVFPFSCRQLQRLGKPVSVRTLFFEHCCWPDGRQRDLFRFILDRPQRVLLTFDGLDEFRFRFAEGVRHCSPTEPTSVQNLLVNLIQGNLMKGSRKVL
SSRPEAVTACLRKYVRKEVGLRGFSQEAVERFMRKHHGDPRVADRIVRLVGATPALRGLCHVPVFSWIVSRCHVELLRLRQGGGGGAPKTMTDMYFLIVR
HLVLRSPLEGDRGPAGGVWRGRLPALLRLGELALGGLGAGCYVFSAGQLQAAGVSAEDLSLGFLVPSKGSAGGAGGSPEARFEFLHVTFQCFLAALYLLL
HGDVAPAAVGHLFHRPRKSRVLELLYPRLCIPAARREEGRPGSLPWGAETVDVQLTASFLAGLLSGTNFAPLAESHGSEALLRKRASARKCLARGIERHF
RSIPPAVPGELKSLHALPAFLWLIKSLYEMQDEGLARRAVRGFEVEHVKLTYCGVGPAECAALAFVLRFLRRPVSLQLDYNSVGDVGLEQLLPCLKVCRA
LYLRDNNISDHGISKLVDQALLCDSLQKLALFNNKLTDDSAHSLAKLLKYKENFLALRLGNNHITAVGAKVLAEGLEGNHSLQFLGLWGNTVGEEGAQAL
ADALHGHRSLKWLSLVGNNVGSVGARALALMLGKNVVLEELCLEENRLNDQDVCSLAEGLKKNSSLKVLKLSNNNITYQGVVSLLQTLKKNDTLKSIWLR
GNTFTPEEIESLSFMDPRLLL

>XP_020840821.1 nucleotide-binding oligomerization domain-containing protein 2 [Phascolarctos
cinereus]
MCTQEFFQAQRSQLVGRLALRSLENFESILDYLLSWEVLTWEDYESVSLPGQPLSTLARRLLDIVWNKGGQSCELLFAAVRKAEGADEQTEPGNQWSQNV
SCPAQDLQRHRPVIVRKIYGHVEGILDLLLEHGFITKYECDEIRLPIFTSSQRARRLLDFAKVKDNGVAQFLLQHIHQLPVLPAPFPDDIAYKKYMAKLK
STVSSQSRFLSTYDGTENLCLEDIYTDNILEIRKDLSVSASSQKCHDIVELPDIFGDSGCINEDADTVLLVGEAGSGKSTLLQQLHLLWATGRHFQDFLF
VFPFTCRQLQSVDKPVSMQTLLFEQCCWPDEGQQEVFQYLLDHPDQVLLTFDGFDEFKFKFTDRETHCSPTDPTSVQSLLFNLLRGNLMKNAKKVLTSRP
HAVSPFLRKYVRKELSLRGFSQGGIELFMRKHHSDPGVADRIVCLVKATSALHGLCHVPVFSWIVSKCHKELLLHGCSSLKTTTDMYLLILQHFVLHASP
EDEGWHPGTTTLRPRLPTILHLGRLALWGLGTCSYVFSAKQLWASGMDEEDLSLGFLVRSKSFSQESSAPMEFLHITFQCFFAAFYLLLGAEVTPSVIRC
LFNHHKKPSRSLAWLFPMSCVQSPEGEEESVTALLQKAEPHNIQITAIFLAGLLSDEHWGLLAECQEPSTPLLQKQSRVRWCLARGLRRHFQSIPPAVPG
EVKSMHAMPEFVWLIRSLYEMQDEHLAREAVCKLEVEHLKVTYCNIGPAECAALAFVLKNLKQPVALQLDYNSVGDVGVEQLLPCLRVCQALYLRDNNIS
DQGICRLVDQALQCDQFQKLALFNNKLTDNCAHSLASLLKYKQNFLALRLGNNHITAVGAKVLAEGLKDNNSLQFLGLWGNAVGDEGAQALASALHDHRS
LKWLSLVGNNIGSLGAQALALMLEKNVSLEELCLEENHLQDEDMCTLVHGLKKNSSLKVLKLSNNSITQQGVSALLQVLQSNTSIKSVWLRGNTFTPEEI
EQLSLMDARLLL

>XP_016283938.1 PREDICTED: nucleotide-binding oligomerization domain-containing protein 2 isoform X1
[Monodelphis domestica]
MYTQEFFQAQRSQLVGRLALRSLENFESVLDYLLSWEVLTWEDYESVSLPGQPLSTLARRLLDIVWNKGGQSCELLFAAVQKAEGADEQTEPLRKWSQNE
SCSVQDLQKHRPTIVRKIYGHVESILNLLLERGFLTKYECDEIRLPIFTSSQRARRLLDFAKVKDNGVAQFLLQHILQLPVLPVPLPDVTACKKYTAKLK
ATVSAQSRFLSTYDGTENLCLENIYTENILEIRKNLSLGVPSQKGHDTLELAEIFNHKGCINEDADTVLLVGEAGSGKSTLLQQLHRLWATGQQFQDFLF
VFPFSCRQLQSMDKPVSVQSLLFEQCCWPNEGQQEVFRYLLDHPDRVLLTFDGFDEFKFRFTERETHCSPTDPTSAQNLLFNLLRGNLMKNTKKVLTSRP
HAICPSLRKYVRKELSLMGFSQDGIELFMRKHHSDAGVADRLVQLVKATSALHGLCHVPVFSWIVSRCHKELLLHGSGFLKTTTDMYLLVLRHFISHASP
EDVAWGLGASTLRPRLPTILRLGRLALWGLGTCSYVFSAEQLRASGVDEEDLSLGFLVPSKSFPPESSAPVEFLHITLQCFFAAFYLLLGADVNPSAIRC
VFGGHKKPHQPLPRLFPMACFQSPEPEEESVLTLLQRAETHNVRITAVFLAGLLSQEHWRLVTECRGTSTPLLQKKSRVRRCLARGLRRHFRSIPRAVPG
EVKSMHAMPEFVWLIRSLYEMQDEHLARKAVSKLEVGHLKVTYCNIGPAECAALAFVLKNLQQPVALQLDYNSVGDIGVEQLLPCLSVCRALYLRDNNIS
DQGICRLVDQALQCDQFQKLALFNNKLTDDCAHSLASLLKYKQNFLALRLGNNHITAVGAKVLAEGLKDNDSLQFLGLWGNTVGDEGAQALACALHDHHS
LKWLSLVGNNIGSVGTQALALMLEKNVSLEELCLEENHLQDEDMCTLVDGLKKNSSLEVLKLSNNRITHRGVSALLQVLESNATIRSIWLRGNTFTPEEI
EQLSLMDSRLLF

>XP_036607169.1 nucleotide-binding oligomerization domain-containing protein 2 [Trichosurus
vulpecula]
MCTQEFFQAQRSQLVRRLALRSLENFESILDYLLSWEVLTWEDYESVSLPGQPLSTLARRLLDIVWNKGGQSCELLFAAVRKAEGADEQAEPEGQWSQNM
SCPAQDLQRHRPLIVRKIYGHVESILNLLLERGFITKYECDEIRLPIFTSSQRARRLLDFVKAKENGVARFLLQHVQQLPVLPAPFPDATACKKYLAKLK
ATVSAQSRFLSTYDGTENLCLEDIYTENILEIQKNLGVRAPSQKCHDTLELLDIFSDSGCINEDADTVLLVGEAGSGKSTLLQQLHHLWATGRHFQDFLF
VFPFTCRQLQSVDKPVSVQTLLFEQCCWPDEGQQEVFQYLLDHPDQVLLTFDGFDEFKFKFTDRETHCSPTDPTSVQSLLFNLLRGNLMKNARKVLTSRP
HAISPFLRKYVQKELSLKGFSQEGIELFMRKHHSDPGVADRIVHLVRAASALHGLCHMPVFSWIVSKCHKELLLHGCGSLKTTTDMYLLILQHFVLHASP
EDGGWRPGTSSLRPWLPTILHLGRLALWGLGTCSYVFSAKQLWESGVEEKDLSLGFLVPSKSFSQESSAPVEFLHITLQCFFAAFYLLLDADVTPSVIRH
LFNHHKKPSRPLAWLFPVSCIQSPEREEASVAELLQKAEPHNIQITAVFLAGLLSQEHWGLLAECQGASTPLLQKQNLTRWCLARGLRRHFQSIPPAVPG
EVKSMHAMPEFVWLIKSLYEMQDERLAREAVCKLEVEHLKVTYCNIGPAECAALAFVLKNLKQPVALQLDYNSVGDIGVEQLLPCLHVCQALYLRDNNIS
```

DQGICTLVDQVLQCDHFQKLALFNNKLTDDCAHSLASLLKNKQNFLALRLGNNHITAVGAKVLAEGLKHNDSLQFLGLWGNAVGDEGAQALASALHDHRS
LKWLSLVGNNIGSLGVQALALMLEKNVSLEDLCLEGNHLQDEDMCTLVHGLKKNSSLKVLRLSNNGITHKGVSALLQVLQSNSSIKSIWLRGNTFTPEEI
EQLSLVDARLLL

>XP_031810335.1 nucleotide-binding oligomerization domain-containing protein 2 isoform X1
[Sarcophilus harrisii]
MCAQEFFQAQRSQLVGRLALRSLENFESILDYLLSWEVLTWEDYESVSLPGQPLSTLARRLLDIIWNKGGQSCELLFAAVRKAEEAEEQTEPPRLWSQNV
ASPAQDLQRHRPVIVRKICGHVEGILDLLLERGFITKYECDEIRLPIFTSSQRARRLLDFAKIKENGVAQFLLQHVHQLPVLPAPFPDAAACRRYRAKLK
ATLSAQSRFLSTYDGSENLCLEDIYTENVLELRKGGGGGGAPGQRGCDTVGLPDIFSDSGCDNEHADTVLLVGEAGSGKSTLLQQLHRLWATGRLFQRFL
FVFPFSCRQLQAVDKPVSVKTLLFEQCCWPDEGQQEVFQCLLDHPERVLITFDGFDEFKFRFTDGETHCSPTEPTSVQNLLFNLLRGNLMKSARKVLTSR
PHAVTPFLRKYVRKELGLRGFSQEGIELFMRRHHSEPGLAERIVRLLKATSALHGLCHVPVFSWIVSRCHKELLQRGCGSPKTTTDMYLLILRHFVMHNS
PGAAAWGPGAAAWGPGVPCLRPWLPTILRLGRLALWGLGTCSYAFSAKQLWARGVEEEDLSLGFLVRSKSFSQESAPSLEFLHITLQCFFAAFYLLLGAD
VRASALRHLFHRREQPGRSLARRFPVSCLRAPEPAEAEEAAASVSGLLQKAEPHNVQITAAFLAGLLSREHRGLVAPGQGAPGPLPRKQSLVRRSLALAL
RRHFRAIPPAVPGEAKSMHAMPEFVWLIRSLYEMQDERLAREAVSKLGVGHLKVTYCNIGPAECAALAFVLKNLQRPVALQLDYNAVGDAGLEQLLPCLP
VCQALYLRDNNISDLGICRLVDQALQCEQFQKLALFNNKLTDDCAHSLATLLKYKQNFLALRLGNNHFTAVGAKVLAEGLKGNDSLQFLGLWGNKVGDEG
ARALASALHDHPSLKWLSLVGNNIGSLGAQALALMLEKNVSLEELCLEENHLQDEDMCVLVRGLKKNSSLKVLKLSNNSITLQGVSVLLQVLEGNATIKS
IWLRGNIFTTEEIEQLSIMDARLLL

>XP_027725377.1 nucleotide-binding oligomerization domain-containing protein 2 isoform X1 [Vombatus
ursinus]
MCTQEFFQAQRSQLVGQLALRSLENFESILDYLLSWEVLTWEDYESVSLPGQPLSTLARRLLDIVWNKGGQSCELLFAAVRKAEGADEQTEPESQWSQNV
SCPVQDLQRHRPVIVRKIYGHVEGILNLLLERGFITKYECDEIRLPIFTSSQRARRLLDFAKVKDNGVAQFLLQHIHQLPVLPAPFPDAIACKKYMAKLK
ATVSAQSRFLSTYDGTENLCLENIYTENILEIRKDLSVSASSQKCHDAVELPDIFSDSDCINEDADTVLLVGEAGSGKSTLLQQLHRLWATGRHFQDFLF
VFPFTCRQLQSVDKMVSMQTLLFEQCCWPDEGQEEVFQYLLDHPDQVLLTFDGFDEFKFKFTDRDTHCSPNDPTSVQSLLFNLLRGNLMKNAKKVLTSRP
HAVSAFLRKYVQKELSLKGFSQEGIELFMRKHHSDPGVADRIVHLVKATSALHGLCHVPVFSWIVSKCHKELLLHGCGSLKTTTDMYLLILQHFVLHASP
DDGGWHPRTTTLQPRLPTILHLGRLALWGLGTCNYVFSAKQLWASGVDEEDLSLGFLVRSKSFSQESSAPMEFLHITFQCFFAAFYLLLGADVTPSVIRC
LFNHHKKPSRSLAWLFPMSCVQSPEGEEESVTALLQKAEPHNIQITAVFLAGLLSHKHWGLLAECQEASTPLLQKQSHVRWCLARGLKRHFQSIPPAVPG
EVKSMHAMPEFVWLIRSLYEMQDEHLAREAVCKLEVGHLKVTYCNIGPAECAALAFVLKNLKQPVALQLDYNSVGDIGVEQLLPCLRICQALYLRDNNIS
DQGICRLVDQALQCDQFQKLALFNNKLTDDCAHSLASLLKHKQNFLALRLGNNHITAVGAKVLAEGLKDNNSLQFLGLWGNAVGDEGAQALASALHDHRS
LKWLSLVGNNIGSLGAQALALMLEKNVSLEELCLEENHLQDEDMCTLVHGLKKNSSLKVLKLSNNSISQQGVSALLQVLQSNTSIKSVWLRGNTFTPEEI
KQLSLMDTRLLL

>XP_004704736.1 nucleotide-binding oligomerization domain-containing protein 2 [Echinops telfairi]
MCTQEGFQARRSQLVSMLVSGSLECFECILDWLLSWEVLSWEDYESLSLLGKPLSHLARHLLDTVWNKGHWSCEKLIAAVQETKNDNHSFELNGLWDPQS
PCPVRDLQTHRPIIVRRLYSHVESVLDRVQEQGFISQYECDGIRLPIFTSSQRARRLLDLATVKKNGLAAFLLQHVRELPVSSAPPFNDAACEKYMSKLK
TTVSAQSRFLSTYDGVENLCLEDIYTENLLELQSDVGMTGGLPQSPATVGLEDLFSRQCHFNDDADTVLVVGEAGSGKSTLLQQLHFLWAAGRAFQEYLF
VFPLSCRQLQCVGKPLSVQMLLFEHCCWPDVGQQDVFQYLLDHPDRVLLTFDGFDEFRFRFTDRERHCSPTEPTSVQNLLFNLLQGNLLKNARKVLTSRP
DAVTACLRKYICAELHLKGFSEEGIELFMRKHHQEPGLADRLVHLLKTTSALHGLCRLPVFSWIVSKCHQELLLLGGGSLRTTTDMYLLILQHFLRHASP
PDSAALSQDPSLLRDRLPTLLHLGHLALWGLGTCCYVFSATQLQAAQVGTEDLSLGFLVRAKSIVPGSSPSLEFLHITFQCFFAAFYLVLSPDFPPTMLR
HLFNCKGPGGSKLARLLPILCIQSTECKENGVTALLQKAEPHNLQITAAFLAGLLSREHWGLLAECQLSQKALLQRHTSARCCLARSLRRHFHSIPPAVP
GEAKSMHAMPSFLWLIRSLYEMQEERLAQEAVHRLDIEHLKLTFCSVGPTECAALAFVLRHLRRPVALQLDHNSVGDIGVEQLLPCLRVCKALYLRDNDI
SDRGVCKLIEHALHCEQLQKLALFNNRLTDGCAHSIAKLLACKHNFLALRLGNNRITAVGAKVLAQGLRDNSSLQFLGFWGNTVGDKGAQALAEALSDHQ
SLKWLSLVGNNIGSVGAQALALMLEKNVVLEELCLEENHLHNEGVCSLAEGLTRNSSLKVLKLSNNHITYVGAEVLLRALEKNDAILEVWLRGNAFSPEE
IQILSHKDIRLLL

>XP_023411378.1 nucleotide-binding oligomerization domain-containing protein 2 isoform X2 [Loxodonta
africana]
MCTQEGFQAKRSQLVGLLVSGSLEGFEGVLDWLLSWEVLSWEDYESLSVLGQPLSHLARRLLDTVWNKGAWGCEQLMAAVQEAQADSRPLELRGLWDPHS
RCPARDLQSHRPAIVRRLYSHVEGVLDLVQERGFVSQYECDGIRLPIFTSSQRARRLLDLATVKTNGLAAFLLQHVQELPVPSALPFEDAACKKYMSKLK
SMVSAQSRFLSTYDGAENLCLEDIYTENVLEVRTNIGMAGPPQQSPATLGLEELFSSQGCLNEDADTVLVVGEAGSGKSTLLQQLHFLWAAGRDFQEFLF
VFPFSCRQLQCVAKPLSVRTLLFEHCCWPDVGQQDVFQFLLDHPDRVLLTFDGFDEFRFRFTDRERHCSPTDPTSVQNLLFNLLQGNLLKNARKVLTSRP
DAVTAFLRKYVCRELHLRGFSEEGIELYMRRHHREPGVADRLICLLKATSALHGLCYLPVFSWIVSRCHQELLLQGGGSLKTTTDMYLLILQHFLLHTSP
PDTDPRGPGPSLLRGRLPTLLRLGHLALWGLGTCCYVFSAKQLQAAQVDTDDIALGFLVRAKSVVLGSSAPLEFLHITFQCFFAAFYLVLSSDLPPTTLR
HLFNCCRTGGSLLARLLPALCIQRSESKESSVVAWLQKAEPHNLQITAAFLAGLLSREHRGLLAECQASEKALLQRHTCARWCLARSLRKYFHSIPPGVP
GEAKSMHAMPGFLWLIRSLYEMQEERLAREAVRGLDVEHLKLTFCGVGPAECAALAFALQHLRQPVALQLDYNSVGDIGVEQLLPCLGVCKALYLRDNNI
SDRGICKLIEHALHFEQLQKLALFNNKLTDGCARSMAELLARKQNFLALRLGNNHITAVGAEVLAQGLRDNCSLQFLGFWGNRVGDKGAQALAEALSDHQ
SLKWLSLVGNSIGSVGAQALARMLEKNMVLEELCLEENHLHDEGVCFLAEGLKRNSSLKVLKLSNNSITYAGAEALLQTLERNNTILEVWLRGNTFSPEE
IETLSHKDTRLLL

>XP_007945192.1 PREDICTED: nucleotide-binding oligomerization domain-containing protein 2
[Orycteropus afer afer]
MCTQEGFQAQRSQLVRLLVSGSLEGFEGVLDWLLSWEVLSWEDYESLSLLGQPLSHLARRLLDTVWNKGAWSCKQLIAAIQEAQANSQPLELHGLWDPQS
PCPARNLQSHRPAIVRKLCSHVEGMLDLVQEQGFISQYECDGIRLPIFTSTQRARRLLDLATVKANGLAAFLLQHVQELPVPSVLSSEDAACKKYMAKLK
TTVSAQTRYLSSYDGAENLCLEDIYTENGLELQTDAGMAGLSQQNPATVGLEELFSPHGPLNEDADTVLVVGEAGSGKSTLLQQLHFLWAAGRDFQEFLF
VFPFSCRQLQCVAKPLSVQMLLFEHCCWPDVGQQDVFQFLLDHPDRVLLTFDGFDEFRYRFTDCERHCSPVNPMSVQNLLFNLLQGNLLKNARKVLTSRP
DAVTAFLRKYVRTELKVKGFSEEGIELYMRKHHRGPGVADRLVHLLKATSALHGLCHLPVFSWIVSKCHQELLLQGGGSLKTSTDMYLLILQHFLLHSSP
PDTTRSSPGPSLLSGRLTTLLHLGCLALWGLGMCCYVFSAKQLQAAQVDTEDISLGFLVRAKSTVPGSSAPLEFLHITFQCFFAAFYLVLSADLPPTILR
HLFNCQRPGGSLLARLLPMLCVPGSEHKEGTVVSLLQEAEPHNLQITAAFVAGLLSQEHRGLLVECQASKKALLRRHACARWCLAHSLRKHFHSIPPAVP
GEAKSMHAMPGFLWLIRSLYEMQEERLAREAACGLDVEHLKLTFCSVGPTECAALAFVLRHLRRPVALQLDYNSVGDVGVEQLLPCLGVCKALYLRDNNI
SDRGICKLIERALHCEQLQKLALFNNKLTDGCAHAMAQLLACKQNFLALRLGNNHITASGAEVLAQGLQDNTSLQFLGFWGNKVGDKGAQALAEALSDHQ

SLKWLSLVGNNIGSVGAKALALMLEKNVVLEELCLEENHLHDEGVCSLAEGLKRNSSLKVLKLSDNFITCAGAEALLQALGRNDTILEVWLRGNTLSPEE
METLSRKDSRLLL

>XP_006873784.1 PREDICTED: nucleotide-binding oligomerization domain-containing protein 2
[Chrysochloris asiatica]
MCTQEHFQAQRSHLVEVLVSGSLEGFESILDHLLSWEVLSWEDYESLSLLGQPLSHLARCLLDTVWNKGAWGCEQLITAVQEVQADSQPHELYGLWGPHS
SCPAQDLQSHRPVIIRRLYSHVEDVLDLAQDQGFISQYECDGIRLPILTSSQRARRLLDLATVKTNGLAAFLLQHVQELPVLSVLPFEGAACKKYMSKLK
TTVSAQSRFLSTYDGVENLCLEDIYTENLLEVQIDVCKAGPPWQRPATLGLQELFGPHGHLNEDADTILVVGEAGSGKSMLLQQLHFLWAAGQDFQEFLF
VFPFSCRQLQCITKPLNVQTLLFEHGCWPDFGQQDIFQFLLDHPDCVLLTFDGLDEFRFRFNDCERHCSPTDPTSVQNLLFNLLQGNLLKNVRKVLTSRP
DAVTAYLRKYVCMELNLKGFSEQGIELYMRKHHREPGVADHLVHLLRTTSALHGLCHLPVFSWIVSKCHQELLLQGGGSLKTSTDMYLLTLQHFLLRTSP
PDLVPRSWGPSLLRDRLPTLVHLGRLALWGLGLCCYVFSATQLQAAQVDSEDIALGFLVRAKSIRPRGSAPLEFLHITFQCFFAALYLILSADLPPTTLR
YLFNCQSLGGFLKARLLPTLCIQSSECKEDNVVALLQKAEPHNLQTTASFLAGLLSQEHRGLLAQCQASEKALLRRHACVRWCLARSIRKHFHSIPPAVP
GEAKSMHAMPGFLWLIRSLYEMQEERLAQEAVRGLDVEHLKLTFCNVGPTECAALAFVLRHLRRPVALQLDHNSVGDTGLEQLLPCLGVCKALYLRDNNI
SDQGISKLIEQALHCEQLQKLALFNNKLTDGCAHSIAKLLACKHNFLALRLGNNHITAVGAEVLAQGLRNNNSLQFMGFWGNKVGDKGAQALAEALSDHQ
SLKWLSLVGNNIGSVGAQALALMLEKNMVLEELCLEENYLHDEGVCFLAEGLQRNSSLKILKLSNNFITCTGAEALLWALERNNTIREIWLRGNTFSPEE
IKMFSHKDTRLLL

>XP_004371651.1 nucleotide-binding oligomerization domain-containing protein 2 isoform X1
[Trichechus manatus latirostris]
MCTQEGFQAQRSQLVGLLVSGSLEGFEGVLDWLLSWEVLSWEDYESLSLLGQPLSHLARRLLDTVWNKGAWGCEQLMAAVQEAQADSQPLELCGLWDPHS
PCPARELQSHRPAIVRRLYSHVEGVLDLTQERGFISQYECDGIRLPIFTSSQRARRLLDLATVKANGLAAFLLQHVQELPVPSALPFKDAACKKYMSKLK
ATVSAQSRFLSTYDGSENLCLEDIYTENVLEVRTNISMAGPSQQSPATLGLEELFSPRGCLNEDADTVLMVGEAGSGKSTLLQQLHFLWAAGRDFQEFLF
VFPFSCRQLQCVAKPLSVRTLLFEHCCWPDVGQQDIFQFLLDHPDRVLLTFDGFDEFRFRFTDGERHCSPTDPTSVQNLLFNLLQGNLLKNARKVLTSRP
DAVTALLRKYVCRELNLKGFSEEGIELYMRKHHREPGVADRLVCLLKATSALHGLCHLPVFSWIVSKCHQELLLQGGGSFKTTTDMYLLILQHFLLHAAP
PDTDRRGLGPSLLRGRLPTLLHLGHLALWGLGTCCYVFSAKQLQAAQVDTDDISLGFLVRAKSVVLGSSAPLEFLHITFQCFFAAFYLVLSSDLPPTTLR
HLFSCCRSGSSPLARLLPALCVQHSERKEDSVAAWLQKAEPHNLQITAAFLAGLLSREHRGLLTECQVSKKALLRRHACARWCLARSLRKHFHSIPPAVR
GEAKSMHALPGFLWLIRSLYEMQEEQLAQEAVRGLEVEHLKLTFCGVGPAECAALAFALQHLRRPVALQLDHNSVGDIGVEQLLPCLSVCKALYLRDNNI
SDRGICKLIEHGLHCEQLQKLALFNNKLTDGCARSVAELLACKQNFLALRLGNNRITAAGAEVLAQGLRDNVSLQFLGFWGNRVGDKGAQALAEALSNHR
SLKWLSLVGNDIGSLGAQALALMLERNVVLEELCLEENHLHDEGVCFLAEGLKRNSSLKVLKLSNNCISCVGAEALLQALERNDTILEVWLRGNTFSPEE
IETLSHKDTRLLL

>XP_037671622.1 nucleotide-binding oligomerization domain-containing protein 2 [Choloepus
didactylus]
MCTQEGFQAQRSRLVGLLISGSLEGFENVLDWLLSWEVLSWEDYESLSLLGQPLSLLARRLLDTVWSKGAWGGEQLIAAIQEAQADGKTPELCGRWDPHS
PHPARELQSHRPAIIRRLYGHVEGVLGLAREQGFISQYECDGIRLPIFTSSQRARRLLDLATVKANGLAAFLLQSALELPVPTALPFEAATCKKYMSKLR
TTVAAQSRYLSTYDGAENLCLEDVYTENILEARTDVSAAGPPQESPAALGLEELFTTHDHLNDAADTVLVVGEAGSGKSTLLQRLHFLWAVGRDFQEFLF
VFPFSCRQLQCLAKPLSLWTLLFEHCCWPDVGQQEVFRFLLDHPERVLLTFDGFDEFRFRFSDHERHCSPTDPTSVQNLLFNLLQGNLLKDARKVLTSRP
EAVSALLRRYVRAELSLKGFSEDGIELYMRKHHREPGVADCLLRLLRATSTLHGLCHLPVFSWIVSKCHRELLQHGGGSLKTTTDMYLLILQHFLLHASP
PDTASHGLGPSFLRGRLPTLLRLGCLALRGLGTCCYVFSAKQLQAAQVDPEDVSLGFLVPVKSQGVSGASAPLEFLHITFQCFFAAFYLVLSADMPPTSL
RHLFDCRGPGSSPLARLLPMLCIQGSECKEGGLAALLQKAEPHNLQITAAFLAGLLSREHRGLLAECQASGKALLRRHACARWCLARSLRQHFHSIPSAV
PGEAKSLHAMPRFLWLIQSLYEMQEERLAREAVRGLSVGHLKLTFCGVGPIECAALAFVLRHLRRPVALQLDHNSVGDIGVEQLLPCLGVCKALYLRDNN
ISDRGICKLIEHALPCEQLQKLALFNNKLTDDCAHSMAKLLACKRNFLALRLGNNRISAVGAAVLAEGLRSNSSLQFLGFWGNRVGDEGAQALAEALGSH
QSLKWLSLVGNNIGSMGAQALALMLERNVVLEELCLEENHLQDEGVGSLAEGLKRNSSLKVLKLSNNCITYLGAEALLQALERNNTILEVWLRGNAFSPE
EAEELSHRDTRLLL

>XP_004450485.1 nucleotide-binding oligomerization domain-containing protein 2 isoform X3 [Dasypus
novemcinctus]
MCTQEGFQAQRSQLLGLLVSGSLEGFESVLDWLLSWEVLSREDYESLSLLGQPLSLLARRLLDTVWSKGAWGCEQLITAVQEAQADSRNPEPHGCWDPHS
PHAARELQSHRPAIIRRLYGHVEGVLGLAQEQGFISQYECDAIRLPIFTSSQRARRLLDLASVKANGLAAFLLRHAQELPVPTALPFEAAPCQKYMSKLR
TTVAAQSRFLSTYDGAENLCLEDIYTDSVLGVRMDVGSAGPLQKSPATLGLGELFHPHGHLNEDADTVLVVGEAGSGKSTLLQRLHSLWAAGRDFQEFLF
VFPFSCRQLQRLDRPLSVRTLLFEHCCWPDVGQQDVLRFLLDHPDRVLLTFDGLDEFRFRFTDRERHCSPNDPTSIQNLLFNLLQGNLLKDARKVLTSRP
DAVSALLRRYVRAELSLEGFSEEGIEMYMRKHHREPGVADCLLRLLRATSALRGLCRLPVFSWMVSKCHRELLQPGGGSPKTTTDMYLLILQHFLLHSSS
PDQAAHGPGPGFLRGRLPSLLHLGCLALRGLGTCCYVFSAKQLQAAQVGPEDISLGFLVPAKGQGVRGGSAPLEFLHVTFQCFFAAFYLVLSADMPPTSL
RHLFGCRRPSSSPLARLLPTLCIQRSEGPEGGVAAWLREAEPHNLQITAAFVAGLLSREHRSLLAECQASGKALLRRHACARWYLARSLRQHFHSIPPAV
PGEAKSVHALPGFLWLIRSLYEMQEEQLAREAVRGLSVGHLKLTFCGVGPAECAALAFVLRHLRRPVALQLDYNSVGDIGVEQLLPCLGVCKALYLRDND
ISDLGICKLIEHALSCEQLQKLALFNNRLTDDCTHSMAKLLACKRNFLALRLGNNHITAVGAAVLAQGLRSNSSLQFLGFWGNRVGDQGAQALAEVLGDH
QSLKWLSLVGNNIGSVGAQALALMLERNMVLEELCLEENHLQDEGVCSLAKALKRNSSLKVLKLSNNRVTYLGAEALLQALERNDTILEVWLRGNTFSPE
EIEKLSHRDSRLLL

>XP_008150595.2 nucleotide-binding oligomerization domain-containing protein 2 isoform X1 [Eptesicus
fuscus]
MEGRDTCTQEAFQAQRSRLVGLLGSGSLEGFESVLDRLLAWEVLSWEDYEGLCVLGQPLSHLARRLLDTVWNKGAWGCEQLLAAVQEARADGQSPALGGH
WDPHSPHPARDLQCHRPAIVRRLYSHVEGVLDLAQVRGFVSPYECDEIRLPIFTSSQRARRLLDLAAVKANGLAAFLLQHVQELPVPLALSVEDAACQKY
TSKLRTTVSAQSRFLSSYDGAENLCLEDIYTENVLELRTEVGAAGAPQTSPAPLGLEALFSAGAPLNADADTVLVVGEAGSGKSTLLQRLHLLWASGRDF
QEFLFVFPFSCRQLQRVARPLSLRALLFEHCCWPDLGQQEVFRFLLAHPDRVLLTFDGFDEFRFRFTEGERHCSPTDPTSVQNLLFNLLQGNLLKNARKL
LTSRPDAVSAFLRKYVRAELHLKGFSEDGIELYLRKRHREPGVADRLVRLLRATSALHGLCHLPVFAWMVSKCHQELLLQGGGSPRTTTDMYLLILQHFL
LHASPPDAAPHGPGPGLLRGKLPTLLHLGRLALWGLGVSCYVFSATQLQAAHVDGADISLGFLVHAKSVAPGGPPPLEFLHITFQCFFAAFYLVLSADVR
PSLLRHLFNCRPTGSSPLARLLPALCVQGPPSEEGSVEAWLQQAEPHNLQITAAFLAGLLSREHRGLLAECQASEKALLRRQACARRCLARSLRKHFCSI
PPALPGEAKSMHALPGFLWLVRSLYELREERLARKAVRGLDVGHLKLTFCSVGPAECAALAFVLRHLRRPVALQLDHNSVGDVGVEQLLPCLGVCKALYL
RDNNLSDRGVCTLAEHALRCEELQKLALFNNKLTDGCAQALARLLECRQNFLALRLGNNRITAAGAQELARGLRANSSLQFLGFWGNEVGDKGAQALAEA

LGDHQSLRWLSLVGNRIGSVGAQALASMLEKNVALEELCLEENQLGDEGVRSLAEGLRRNSSLKVLKLSNNRVTYRGAEALLQALERNDTVREVWLRGNA
FSPEEMETLGRRDARLLL

>XP_014303493.1 nucleotide-binding oligomerization domain-containing protein 2 isoform X1 [Myotis lucifugus]
MEGHDTCTQQAFQAQRSQLVGLLGSGSLEAFESVLDWLLSWDVLSWEEYEGLHVLGQSLSHSARRLLDTVWNKGAWGCEQLLAAVQEAQADGRSPELHGH
WDPHSPHPARDLQCHRPAIVRRLYSHVEGVLDLAQARGFLSPYECDEIRLPIFTSSQRARRLLDLATVKANGLAAFLLQHVQELPVPLALSLEDAACRKY
TSKLRTTVSAQSRFLSSYDGAENLRLEDIYTENVLELRTEAGAPQTSPAPPGLDALFRSGSPRNADADTVLVVGEAGSGKSTLLQRLHLLWASGRDFQDF
LFVFPFSCRQLQRLAKPLSVRALLFEHCCWPDLGQEEVFRFLLAHPERVLLTFDGFDEFRFRFTDGERHCSPTAPTSVQNLLFNLLQGNLLKNARKLLTS
RPDAVSAGLRRYVRTELHLRGFSEDGIELYLRKRHREPGLADRLVRLLRATSALHGLCHLPVFAWMVSKCHQELLLQDGGSPRTTTDMYLLILQHFLLHA
SPPDAAPHGPGPGLLRGRLPTLLHLGRLALWGLGVSCYVFSAQQLQAAHVDGADVSLGFLVHAKSAAPGSPPPLEFLHITFQCFFAAFYLVLSADVRPSS
LRHLFNCRPTGSSPLARLLPALCVQGPPREEGGVEAWLQQAEPHNLQITAAFLAGLLSREHRGLLAECQASEKALLRRQACARRCLARSLRKHFCSIPPA
LPGEAKSMHALPGFLWLVRSLYELREERLARKAVRGLDVGHLKLTFCSVGPAECAALAFVLRHLRRPVALQLDHNSVGDVGVEQLLPCLGVCKALYLRDN
NISDRGMCTLAEHALRCEELQKLALFNNKLTDGCAQALARLLECRQNFLALRLGNNHITEAGAQVLARGLRANTSLQFLGFWGNEVGDKGAQALAEALGD
HQSLRWLSLVGNRIGSVGARALASMLEKNMALEELCLEENQLGDEGVCSLAEGLRRNPSLKVLKLSNNCVTYRGAEALLRALERNDTVREVWLRGNAFSP
EEMETLGHRDARLLL

>XP_032985452.1 nucleotide-binding oligomerization domain-containing protein 2 [Rhinolophus ferrumequinum]
MCAQEAFQAQRSQLVELLVSGSLEGFESILDWLLSWEVLSWEDYEGLSLLGQPLSHLARRLLDTVWNKGAWGCEQLIAAVQEAQADSQSPKLCDHWDPHS
PHPAQDLRSHRPAIVRRLYSHVEGVLDLAKERGFISLYECDEIRLPIFTSSQRARRLLDLAMVKANGLAPFLLQHVEELPVPKALPFEDAACKKYMSKLR
TTVSAQSRFLSTYDGAESLCLEEIYTENVLEIRTEVGMAGPPQNCPATLGLEGLFSSHGLNKDADTVLVVGEAGSGKSTLLQQLHLLWAAGRDFQDFLFV
FPFSCRQLQCVAKPLSMQALLFEHCCWPDLGQQDVFQFLLEHPDRVLLTFDGFDEFKFRFTDRERHCSPTHPTSVQNLLFNLLQGNLLKNARKVLTSRPD
AVSACLRKYVRMELTLKGFSEDGIELYLRKRHRDPGVADRLISLLKATSALHGLCHLPVFSWMVSKCHQELLLQGGGSPTTTTDMYLLILQHFLQHTSPP
DSTPPHLGAGLLRGRLPTLLHLGRLALWGLGMCCYVFSAKQLQAAHVDSEDISLGFLVRAQRAVSESTAPLEFLHITFQCFFAALYLVLSADMPPSSLRY
LFNCHPPESSLLARLLPALCVPGSRCKEDSVAVLLQEAEPHNLQITAAFLAGLLSQEHRSLLAECQASEKALLRRQDCARRCLAHSLHKHFQSIPPAVPG
EAKSMHAMPGFVWLIRSLYEMQEERLAREAVRRLNVEHLKLTFCSVGPAECAALAFVLQHLRWPVALQLDHNSVGDIGVEQLLPCLGVCKALYLRDNNIS
DRGICKLIEHALHCEQLQKLALFNNKLTDGCAHSMARLLECRRNFLALRLGNNHITAVGAQVLAQGLRANASLQFLGFWGNKVGDKGAQALAEALGDHQS
LRWLSLVGNNIGSVGAQALALMLEKNVALEELCLEENHLQDEGVCSLAEGLKRNSSLKVLKLSNNCVTYLGAEALLQALARNDTILEVWLRGNTFSPEEI
EKLSHRDTRLLL

>XP_024407323.1 nucleotide-binding oligomerization domain-containing protein 2 [Desmodus rotundus]
MCTQEAFQAQRSQLVELLVSGSLEGFESVLDWLLSWEVLSWEDYEGLSLPGQPLSHLARRLLDTVWNKGAWGCEQLIAAVQEARVDRQSPKLRGHWDPHS
PHPARDLRCRRPAIVRRLYGHVEGVLELAQKRGFISPYECDEIRLPIFTSSQRARRLLDLAMVKANGLAAFLLQHALELPVPLTLPFEDAAACKKYISKL
RTTVSAQSRFLSTYDGAENLCLEEIYTENVLEIRTEVGVARPLKQSPATLGLEGLFGASGHLNEDADTVLVVGEAGSGKSTLLQQLHLLWAAGRDFQEFL
FVFPFSCRQLQCVAKPLSVQTLLFEHCCWPDLGQQDVFQFLLDHPHRVLLTFDGFDEFRFRFTDRERHCSPTDPTSVQNLLFNLLQGNLLKNARKVLTSR
PDAVSAFLRKYVRMELHLKGFSEDGIELYLRKRQREPGVADRLIRLLKETSTLHGLCHLPVFAWMVSKCHQELLLQGGGPPKTTTDMYLLFLQHFLLHAS
PPDSARHTLGPSLLRGRLPTLLHLGRLALWGLGMSCYVFSAQQLQAAQVNEEDISLGFLVHAQGVVPGSTPRLEFLHITFQCFFASLYLALSVNVTPSSL
RHLFSCGGTGSSPLARLLPTLCVQGPRPREGSVEALLQEAEPHNLQITAAFLAGLLSQEHRGLLAECQASEKALLRRQACARWCLARSLQKHFHSIPPAL
PGEAKSMHAMPGFVWLIRSLYELREERLAREAVHGLRVEHLKLTFCGVGPTECAALAFVLQHLRRPVALQLDHNSVGDVGVEQLLPCLHVCKALYLRDNN
ISDRGICKLLEHALHCEALQKLALFNNKLTDGCAQSVARLLECRQNFLALRLGNNHITSVGAQVLARGLRANTSLQFLGFWGNKVGDKGAQALAEALGDH
QSLRWLSLVGNDVGSAGAKALALMLEKNVALEELCLEENHLQDEGVCSLAEGLTRNSSLKVLKLSNNCVTYRGAQALLQALERNSTILEVWLRGNTFSPE
EIEQLSHRDTRLLL

>XP_037371669.1 nucleotide-binding oligomerization domain-containing protein 2 isoform X1 [Talpa occidentalis]
MCTQEAFQARRSQLVGLLISGSLEGFESVLDWLLSWDVLSWEDYEGLRLVGQPLSHLARRLLDTVWNKGTWGCEQLVAAVGEAQADSQAPELPDRWDPRS
PQPARDLQSHRPAIVRRLYSHVEHVLDVARERAFISRYEYDEIRLPIFTASQRARRLLDLATVKVNGLAAFLLQHVQELPAPLALPFEDAACKKYMSKLR
TTVSAQSRFLSTYDRAENLCLEEIYTENVLEIHTDVGVAGALQRSPTLLGLEELFSPHDHTNQDADTVLVVGEAGSGKSTLLQQLHLLWALGRDFQEFLF
VFPFSCRQLQCVAKPLSVQTLLFEHCCWPDLGQQDVFRFLLDHPDRVLLTFDGFDEFKFRFSDSQRHCSPTDPTSVQNLLFNLLQGNLLKGARKVLTSRP
DAVSALLRKYVRMELSLKGFSEEGIELYLRRCHREPEVADRLISLLKATSALHGLCHLPVFSWMVSKCHQELLLQGGESLKTTTDMYLLILQHFLQHASP
LDTAAQGLEPRLLRARLPTLLHLGQLALQGLGTCCYVFSAQQLQAAHVDMEDIALGFLVRAGRALPGSTGPLEFLHITFQCFFAAFYLVLSADMPPSSLR
HLFSCHRPGSSLLARLLPRMCVPGAGPKEGSVAALLQGVDPHNLQITAAFLAGLLSQEHCGLLAECQVSEKALLRRQACARGCLARSLHRHFHSIPPAVP
GEAKSMHAMPGFVWLIRSLYEMQEERLAREAVRGLDVGHIKLNFCSVGPTECAALAFVLRHLRRPVALQLDHNSVGDIGVQQLLPCLGVCKALYLRDNNI
SDRGICKLVERALHCEQLQKLALFNNRLTDGCAHAMARLLSCKQNFVALRLGNNHITASGAEVLAQGLRANASLQFLGFWGNKVGDKGAQALAEALGDHQ
SLRWLSLVGNSIGSVGAQSLALMLEKNVALEELCLEENHLGDEGVCSLARGLKRNSSLKVLKLSNNCVTCRGAEALLQALERNDAIREVWLRGNAFSPED
VEQLCRRDARLLL

>XP_004600730.1 PREDICTED: nucleotide-binding oligomerization domain-containing protein 2 [Sorex araneus]
MCTQQAFQAQRGQLVGQLISGSLEGFESVLDWLLAWDVLSWEDYEGLSLLGQPLSHLARRLLDTVGKKGAWGCERLIEALREAQAQDQAPELPAGWNPHT
PHDLQSHRPAVIRRLYSHVEGVLTQAQERGFVSQYECDGIRLPIFTSAQRARRLLDLATVKSNGLATFLLHRIQELPVLSSLSFEDAACKKYMSKLRTTI
SAQSRFLSTYDGTENLCLEEVYTENALEIRTEPGQAGPRQGSPSLLGLEDLFSARGHLNPDADTVLVVGEAGSGKSTLLQQLLLLWAAGRDFQEFLFVFP
FSCRQLQRVAKPLSVQTLLFEHCCWPDVGRPDVFQFLLDHPDRLLLTFDGFDEFRFRFSDCERHCSPTDPTSVQNLLFNLLQGNLLKNARKVLTSRPDAV
SALLRKYVRAELNLKGFSEEAIALYLRKRHHEPGVADRLISLLQATPTLHGLCHLPVFSWMVSKCHQELLLQGGGALKTTTDMYLLILQHFLLHASPVAT
AAPRLAPCPLRDWLPTLLHLGRLALWGLGTCCYVFTDKQLREASVEAADMTLGFLVHAQRAGPSGGASLEFLHITFQCFFAAFYLVLGADVPPSSLRHLF
RCGRPGGWLRGRLLPALCVPGSGTREGSVAALLQEAETHNLQMTAAFLAGLLSREHRDLLAAAEGRASGKALLQRQACARRCLARSLHRHFRGIPAAVPG
EAKSMHAMPNFVWLIRSLHEMQEERLAREAVRGLDVGHLKLTFCGMGPAECAALAFVLRHLRQPVALQLDHNSVGDIGVEQLLPCLGVCKALYLRNNNIS
DRGICLLVEHALRCEPLQKLALFNNRLTDGSARALARLLACKRNFLSLRLGNNHFTAAGAQVLAEGLRGNTSLQFLGFWGNKVGDHGAQALAEALGKHEN

LKWLSLVGNNIGRVGARALALMLEQTMSLEELCLEENQLEDEGVCSLAEGMKRNSSLKILKLSNNGVTHLGATALLQALEENATLQEVWLRGNAFSPEEL
ERLSRQDTRLLL

>XP_007538456.2 PREDICTED: nucleotide-binding oligomerization domain-containing protein 2 [Erinaceus europaeus]
MGVGREMCTQEAFQAQRSELLGLLVSGSLEGFESVLDWLLSWGVLSWEDYEGLSLLGQPLSRSARHLLDT
VWSKGAWGCERLIAAVNEAQADSQVLELPSCWNPHSSHPARDLQSHRPAVIRRLYSRVEGVLEQARQQGFISRYECDEIRLPIFTASQRARRLLDLATVK
ANGLAAFLLQHVQELPVLLAPPFEDAACKKFTSKLRSTLSAQSRFLSTYDGSENLCLEEIYTENVLEIRTEVGLAGSPQKSPAILGLEDLFSTRGQLNAD
ADTVLVMGEAGSGKSTLLQRLHLLWATGRDFQEFLFVFPFSCRQLQCVAGPLSMQTLLFQHCCWPDLGQQDVFQFLLDHPDRVLLTFDGFDEFRFRFSDQ
ERHCSPTDPTSVQNLLFNLLQGNLLKNACKVLTSRPAAVSSLLRKYVRLELQLKGFSEEGIKVYLRKCHRQPGVAERLIGLLRATSALHGLCHLPVFSWM
VSKCHQELLLQGGGSPKTTTDMYLLILQHFLLHACQPDLVAHSVGPCLLKARLATLLHLGRLALWGLGHCCYVFSAGQLQAAHVDAEDIALGFLVRARRV
VPGSLAPLEFLHITFQCFFAAFYLVLSTDAPPSLLRQVFSCRRPGSPLVARLLPALCVPGPGHREGSVAALLQEAEPHNLQITAAFVAGLLAQEHQGLLA
ECQASEKALLRRQACARWCLARSLQRHFHSIPPAVPGEAKSMHAMPGFLWLIRSLYELQEERLAREAVRGLDVGHLKLTFCGVGPVECAALAFVLRHLRR
PVALQLDHNSVGDIGVEQLLPCLRVCKALYLRNNNISDRGVCKLVEHALHCEQLQKLALFNNKLTDGCTHAMARLLTCRQNFLSLRLGNNNITAAGAEVL
AGGLRANASLQFLGFWGNKVGDKGAQALAEALGDHTNLKWLSLVGNNIGSVGAQALAVMLEKNAVLEELCLEENHLQDEGVCSLTDGLQRNASLKILKLS
NNGITDLGAEALLQALERNNTIQEVWLRGNSFSPEEMEKLSHRDARLLL

>XP_036691091.1 nucleotide-binding oligomerization domain-containing protein 2 isoform X1 [Balaenoptera musculus]
MYTQDDFQAQRSQLVELLVSGSLEGFESVLDWLLSWEVLSWEDYEGLSLLGQPVSHLARRLLDTVWNKGAWGCELLTAAVQETQADRQPPELPGCWEPHS
PHPARDLQSHRPAIVRRLYGHVEGVLDLTQARGFISQYECDEIRRPIFTSSQRARRLLDLATVKANGLAAFLLQCVQELPVPLALPFEDAACKRYMSKLR
TTVLAQSRFLSTYDGTENLCLEEIYTENALEIRMEADMAGPLQQSPATLGLEELFSTRGHLNEDADTVLVVGEAGSGKSTLLQQLHLLWASGRAFQEFIF
VFPFSCRQLQCLAKPLSVWTLLFEHCCWPDLGQQDVFQVLLDHPERILLTFDGFDEFKFRFMDRERHCCPTAPTSVQSLLFNLLQGNLLKNARKVLTSRP
DAVSASLRKHVRTELSLKGFSEEGIELYLRRRHREPGVADRLIRLLRATSALHSLCHLPVFSWMVSKCHQELLLQGGGSPKTTTDMYLLILQHFLLHASP
PDSAAHGLGAGLLRGRFPTLLRLGRLALWGLGTCCYVFSTKQLQAAHVDSEDVSLGFLVRAKRVVPGSTAPLEFLHVTFQCFFAAFYLALSADTPPSSLR
HLFNGHGPGCSPLARVLPKLCVRGSGCKEGSVAALLQGAELHNLQITAAFLAGLLSQERQGLLAECQVSEKALLCRQACTRQCLARSLRKHFRSIPPAVP
GEAKSMHAMPSFIWLVRSLYEMQEERLAWEAVRRLDVGHLKLTFCSVGPAECAALAFVLRHLRQPVALQLDHNSVGDIGVEQLLPCLGVCKALYLRDNNV
SDRGICKLTEHALRCERLQKLALFNNKLTDGCAHSMAKLLACKQNFLALRLGNNHITAAGAEVLAQGLRANTSLQFLGFWGNKVGDRGAQALAEALGDHQ
SLRWLSLVGNNIGSVGAQALALMLEKNVALEELCLEENHVQDEGVCCLAKGLEKNSSLKVLKLSNNRITSLGAEALLQALEKNDTILEVWLRGNTFSPEE
IEKLSHRDTRLLL

>XP_019788051.1 nucleotide-binding oligomerization domain-containing protein 2 isoform X1 [Tursiops truncatus]
MYTQDAFQAQRSQLVELLVSGSLEGFESVLDWLLSWEVLSWEDYEGLSLLGQPVSHLARRLLDTVWNKGAWGCELLTAAVRETQADRQPPELPGCWEPHS
PHPARDLQSHRPAIVRRLHGHVEGVLDLTQARGFISRYECDEIRRPIFTSSQRARRLLDLATVKANGLAAFLLQCVQELPVPLALPFEDAACKRYLSKLR
TMVSAQSRFLSTYDGTESLCLEEIYTENVLEIRTEAGMAGPLQQSPATLGLEELFSTRGHLNEDADTVLVVGEAGSGKSTLLQQLHLLWASGRAFQEFIF
VFPFSCRQLQCLAKPLSVWTLLFEHCCWPDLGQQDVFQVLLDHPERILLTFDGFDEFRFRFTDRDRHCCPTAPTSVQSLLFNLLQGNLLKNARKVLTSRP
DAVSASLRKHVRTELNLKGFSEEGIELYLRKRHREPGEAERLICCLRATSALHSLCHLPVFSWMVSKCHRELLLQGGVSPKTTTDMYLLILQHFLLHASP
PDSAAHGLGAGLLRGSLLTLLHLGRLALWGLGTCCYVFSPRQLQAAHVDSEEVSLGFLVRAKTVVPGSTAPLEFLHITFQCFFAAFYLALSADTPPSSLR
HLFNCHGPGCSPLAGVLSKLRMRDSGCKEGSVAALLQGAEPHNLQITAAFLAGLLSQEHRGLLAECQVFEKALLWRQACTRRCLARSLRKHFRSIPPAVP
GEAKSVHAMPSFIWLIRSLYEMQEERLAREAVRRLDVGHLKLTFCSVGPAECAALAFVLRHLRQPVALQLDHNSVGDMGVEQLLPCLGVCKALYLRDNNV
SDRGICKLTEHAVRCEQLQKLALFNNKLTDGCAHSMAKLLACKQNFLALRLGNNHITAAGAEVLAQGLRANASLQFLGLWGNKVGDRGAQALAEALGDHR
SLRWLSLVGNDIGSVGAQALALMLEKNMALEELCLEENHVQDEGVCCLAKGLEKNSSLKVLKLSNNGITSLGAEALLQALEKNDTILEVWLRGNTFSPEE
TEKLSHRDTRLLL

>XP_023492719.1 nucleotide-binding oligomerization domain-containing protein 2 isoform X1 [Equus caballus]
MCTQEAFQAQRSQLVGLLVSGSLEGFESVLDWLLSWDVLSWEDYESLSLVGQPLSHLARHLLDTVWKKGA
WGCEQLVAAVREAQADSQSPELPGHWDPHSPQPAQDLQSHRPAIVRRLYSHVEGVLDLAHERGFISRYECDEIRLPIFTSSQRARRLLDLATVKANGLAA
FLLQHVQELPVLLALPFQDAACKKFMSKLRTTVSAQSRFLNTYDGAENLCLEEIYTENVLEIRTELGMAGPPQQSPATLGLEELFSTRGHLNKDADTVLV
VGEAGSGKSTLLQQLHLLWAAGRDFQEFLFVFPFSCRQLQCLAKPLSVRALLFEHCCWPDLGQQDVFQFLLDHPDRVLLTFDGFDEFRFRFTDGERHCSP
TDPTSVQNLLFNLLQGNLLKNARKVLTSRPDAVSALLRKYVHTEFTLKGFSEEGIELYLRKCHREPGLADRLISLLRATSALHGLCHLPVFSWMVSKCHQ
ELLLQGGGSPKTTTDMYLLILQHFLLHASPPDSAPGGLEPGLLRGRLPTLLHLGQLALWGLGTCCYVFSAKQLQAAHVDSEDISLGFLVRAKRAVPGSAP
PMEFLHITFQCFFAAFYLVLSADVPPSSLRYLFSCGRPGSSLLARLLPAMCVQGSRCKEDSVEALLQEAEPHNLQITAAFLAGLLSREHRGLLAKCQGSE
TALRQRQACARWCLARSLHKHFHSIPPAVAGEAKSMHAMPGFIWLIRSLYEMQEERLAQEAVRGLDVGHLKLTFCSVGPAECAAMAFVLRHLRRPVALQL
DHNSVGDVGVEQLLPCLSVCTALYLRDNNISDRGICQLIERALHCEQLQKLALFNNKLTDGCAHSMARLLMCKQNFLALRLGNNHITAAGAVVLAQGLRA
NTSLQFLGFWGNKVGDKGAQALAEALGNHPSLKWLSLVGNSIGSAGAQALAVMLEKNVALEELCLEENHLQDEGVCSLAEGLQRNSSLKVLKLSNNCITY
RGAEALLQTLERNDTILEVWLRGNTFSPEEIEKLSHRDARLLL

>XP_032181200.1 nucleotide-binding oligomerization domain-containing protein 2 isoform X1 [Mustela erminea]
MCTQEAFQAQRNQLVGLLVSGSLEGFESILDWLLSWEVLSWEDYEGLSLLGQPLSHLARRLLDTVWNKGTWGCEQLIAAIQEAQTDYQARELSSCWDPHS
PHPARNLQSHRPAIVRRLHGHVEGVLDLAWEHGFISQYECDEIRLPIFTSSQRARRLLDLATVKANGLAAFLLRHVQKLPVSLALPFEDAACKKYMSKLR
TTISAQSRFLSTYDGAENLCLEEIYTENVLEIRTERGPAGLPQKSPVTLGLEELFGTRGHLNEEADTVLVVGEAGSGKSTLLQQMHLLWASGRDFQEFLF
VFPFSCRQLQCVAKPLSVQTLLFEHCCWPDLGQQEVFQFLLDHPNRVLLTFDGFDEFKFRFSDRERHCSPTDPTSVQNLLFNLLQGNLLKNARKVLTSRP
DAVSALLRKYLRLELHLKGFSEEGIELYLRKCHREPGVADRLVRLLKATSPLHGLCHLPVVSWMVSKCHQELLLHGGGSPKTTTDLYLLILQHFLLHASP
PDSVPCGLAPGLLQGRLPSLLHLGQLAVWGLGTCCYVFSDKQLQAAHIDSEDISLGFLVHAKSVVHGGTAPLEFLHITFQCFFAALYLVLSADLSPSLLR
QLFSCHGPRSSLLARLLPTMCVPRSEHQEGSTAALLQEAEPHNLQITAAFLAGLLSREHQGLLAGSQVSEKALLQRQACARSCLSCSLHKHFHSIPPAVP
GEAKSMHAMPGFIWLIRSLYEMQEERLAREAVRGLKVGHLKLTFCGVGPAECAALAFVLRHLRRPVALQLDHNSVGDVGVEQLLPCLNVCKALYLRDNNI
SDRGVCKLVERALHCEQLQKLALFNNKLTDGCAPSMARLLSCKRNFLALRLGNNHITATGARVLAEGLRANASLQFLGLWGNKVGDEGAQALAEALGDHQ

SLRWLSLVGNNIGSTGAQALALMLEKNMALEELCLEENRLQDEGVCSLAEGLEKNSSLKVLKLSDNSITYFGAEAVLRALERNDTILEVWLRGNTFSAEE
VERLGQRDTRLLL

>NP_001273968.2 nucleotide-binding oligomerization domain-containing protein 2 [Canis lupus
familiaris]
MCTQEAFQTQRSQLVGLLVSGSLEGFESILDWLLSWEVLSWEDYEGLSLLGQPLSYLARRLLDTVWNKGTWGCEQLVAAVREAQTDCQALELSSCWDPHS
PHPARDLQSHRPAIVRRLYSHVEDVLNLAWEQGFISQYERDEIRLPIFTSSQRARRLLDLATVKANGLAAFLLRHVQELPVPLAMPSEDAACKKYMSKLR
TTISSQSRFLSTYDGAENLCLEEIYTENVLEIRTEMGLARSPQKSPATLSLEELFSTCGHLNEDADTVLVVGEAGSGKSTLLQRMHLLWASGRDFQEFLF
VFPFSCRQLQCVAKPLSVQMLLFEHCCWPDFGQQEVFQFLLDHPNRVLLTFDGFDEFRFRFSDHERHCSPTDPTSVQNLLFNLLQGNLLKNARKVLTSRP
DAVSALLRKYLRLEINLKGFSEEGIELYLRKCHREPGVADRLIRLLKTTSALHGLCHLPVVSWMVSKCHQELLLHGGGSPKTSTDMYLLILQHFLLHASP
PDSVPHSLGSHLLRGRLPTLLHLGWLALWGLGMCCYVFSAKQLQAAHIDDEDISLGFLVHAKTVGPGSTTPLEFLHITFQCFFAALYLVLSTDVSPSLLR
QLFICHGPRSSLLARLLPTTCVPRSERKEGSLAALLQEAEPHNLQITAAFLAGLLSREHRGLLAECQANEEALLRLQGRAQGCLSRSLHQHFRSIPPAVP
GEAKSMHAMPGFLWLIRSLYEMQEERLAREAVRGLTVGHLKLTFCGVGPPECAALAFVLRHLRRPVALQLDHNSVGDIGVEQLLPCLSVCKALYLRDNNI
SDRGICKLIEHALHCEQLQKLALFNNKLTDGCAHSMARLLACKQNFLALRLGNNHITAAGAQALAEGLRANTSLQFLGFWGNKVGDEGAQALAEALGDHQ
SLRWLSLVGNDIGSVGARALALMLEKNVALEELCLEENHLQDEGVCSLAKGLERNSSLKVLKLSNNCITYLGAEGLLQALEKNDTILEVWLRGNTFSLEE
MERLSQKDTRLLL

>XP_019675297.2 nucleotide-binding oligomerization domain-containing protein 2 isoform X1 [Felis
catus]
MTACYEMCTQEALQAQRSQLVGLLVSGSLEGFESILDWLLSWEVLSWEDYEGLSLLGQPLSQLARCLLDTVWKKGAWGCEQLIAAVREAQTGCQAPELSC
RWDPHSPHPAHDLQSHRPAIVRRLYSHVEGVLDLAQERGFISRYECDEIRLPVFTSSQRARRLLDLATVKANGLAAFLLRHVQELPVPVALPFGDAACEK
YMSKLRTTLSAQSRFLSTYDGADNLCLEEVYTENVLEIRTELGVAGPPPKSPATLGLGELFSTCGHLNEDADTVLVVGEAGSGKSTLLQRVHLLWTTGRH
FREFLFVFPFSCRQLQRVAKPLSVQTLLFEHCCWPDVGRQDVFQFLLDHPNRVLLTFDGFDEFRFRFTDRGERHCSPTDPTSVQNLLFNLLQGNLLKNAR
KVLTSRPDAVSALLRKYLRVELNLKGFSEEGIELYLRKRHREPGVADRLIRMLRATSALHGLCHLPVITWMVSKCHQELLLPGGGSPRTTTGMYLLIVQH
FLLRASPPDAAPRSPGPGLLRGRLPALLHLGWLALWGLGRCCYVFSSRQLQAAHVDGDDVSLGFLVRAKSVAPGSVAPLEFLHITFQCFFAAFYLVLSAD
VPPSSLRRLFRCHTPGGSLLARLLPAACVRRPAREEGGVAALLQEAEPHNLQITAAFLAGLLSREHRGLLAEGQASEKALLRRRVCARWCLSRSLHKHFH
AIPPAVPGEAKSMHAMPGFVWLSRSLYEMQEERLAREAVRGLKVGHLKLTFCGVGPAECAALAFVLRHLRRPVALQLDHNSVGDVGVEQLLPCLDVCKAL
YLRDNNISDRGICKLVEHALHCEQLQKLALFNNKLTDGCAHSVARLLACRQNFLALRLGNNHITASGAQVLAEGLRANASLQFLGFWGNKVGDEGAQALA
EALGDHQSLKWLSLVGNNIGSVGAQALALMLEKNVTLEELCLEENHLRDEGVCSLAKGLQRNSSLKVLKLSNNHITDLGAEVLLQALERNDTILEVWLRG
NTFSPEETERLSQRDTRLLL

>NP_001002889.1 nucleotide-binding oligomerization domain-containing protein 2 [Bos taurus]
MCAQDAFQTQRSQLVELLVSGSLEGFESILDRLLSREVLSWEDYEGLSLVGQPISHLARRLLDTIWNKGTWGCEQLTAAVREAQADSQPPELPSSWDPHS
PHPARDLQSHRPAIVRRLYGHVEGVLDLTQQRGFISQYETDEIRRPIFTSSQRARRLLDLATVKANGLAAFLLQCIQELPVPLALPFEDAACKKYVSKLR
TVISAQSRFLSTYDGAENLCLEEVYTENVLEIQMEVGMAGPSQQSPTTLGLEELFSTRDHFNKEADTVLVVGEAGSGKSTLLQQLHLLWASGRAFQEFLF
VFPFSCRQLQCLVKPLSMRTLLFEHCCWPDLGPQDVFQVLLDHPERILLTFDGFDEFRFRFTDQERHCCPTAPTSVQSLLFNLLQGNLLKNARKVLTSRP
SAVSASLRKHVRTELSLKGFSEEGIELYLRKRHREPGVADRLLCLLRATSALHGLCHLPVFSWMVSKCHEELLLQGRGSPKTTTDMYLLILRHFLLHASP
LPLATHGLGPSLIQGRLPTLLHLGRLALWGLGTCCYVFSAKQLQAAHVDSEDLSLGFLVLAKRVVPGSTAPLEFLHITFQCFFAAFYLALSADTPPSSLR
HLFQDHRPESSPLARVLPKLFLRGSRCREGSVAALLQGAEPHNLQITGAFLAGLLSQEHRSLLAECQASETALLRRWDCVRRCLTRSLREHFRSIPPALP
GEAKSMHALPGFLWLIRSLYEMQEERLAREAVCRLNVGHLKLTFCGVGPAECAALAFVLRHLRRPVALQLDHNSVGDIGVEQLLPCLGVCKALYLRDNNI
SDRGICKLVEHALRCEQLQKLALFNNKLTDGCAHSMARLLACKQNFLALRLGNNHITAAGAEVLAQGLRTNNSLQFLGFWGNQVGDEGAQALAAALGDHQ
SLRWLSLVGNNIGSVGAQALALMLEKNMALEELCLEENHVQDEGVCFLAKGLARNSSLKVLKLSNNHISSLGAEALLRALEKNDTILEVWLRGNTFSPEE
IEKLSHQDTRLLL

>NP_665856.2 nucleotide-binding oligomerization domain-containing protein 2 [Mus musculus]
MCSQEEFQAQRSQLVALLISGSLEGFESILDWLLSWDVLSREDYEGLSLPGQPLSHSARRLLDTVWNKGVWGCQKLLEAVQEAQANSHTFELYGSWDTHS
LHPTRDLQSHRPAIVRRLYNHVEAMLELAREGGFLSQYECEEIRLPIFTSSQRARRLLDLAAVKANGLAAFLLQHVRELPAPLPLPYEAAECQKFISKLR
TMVLTQSRFLSTYDGSENLCLEDIYTENILELQTEVGTAGALQKSPAILGLEDLFDTHGHLNRDADTILVVGEAGSGKSTLLQRLHLLWATGRSFQEFLF
IFPFSCRQLQCVAKPLSLRTLLFEHCCWPDVAQDDVFQFLLDHPDRVLLTFDGLDEFKFRFTDRERHCSPIDPTSVQTLLFNLLQGNLLKNACKVLTSRP
DAVSALLRKFVRTELQLKGFSEEGIQLYLRKHHREPGVADRLIQLIQATSALHGLCHLPVFSWMVSRCHRELLLQNRGFPTTSTDMYLLILQHFLLHASP
PDSSPLGLGPGLLQSRLSTLLHLGHLALRGLAMSCYVFSAQQLQAAQVDSDDISLGFLVRAQSSVPGSKAPLEFLHITFQCFFAAFYLAVSADTSVASLK
HLFSCGRLGSSLLGRLLPNLCIQGSRVKKGSEAALLQKAEPHNLQITAAFLAGLLSQQHRDLLAACQVSERVLLQRQARARSCLAHSLREHFHSIPPAVP
GETKSMHAMPGFIWLIRSLYEMQEEQLAQEAVRRLDIGHLKLTFCRVGPAECAALAFVLQHLQRPVALQLDYNSVGDVGVEQLRPCLGVCTALYLRDNNI
SDRGARTLVECALRCEQLQKLALFNNKLTDACACSMAKLLAHKQNFLSLRVGNNHITAAGAEVLAQGLKSNTSLKFLGFWGNSVGDKGTQALAEVVADHQ
NLKWLSLVGNNIGSMGAEALALMLEKNKSLEELCLEENHICDEGVYSLAEGLKRNSTLKFLKLSNNGITYRGAEALLQALSRNSAILEVWLRGNTFSLEE
IQTLSSRDARLLL

>NP_001280486.1 nucleotide-binding oligomerization domain-containing protein 2 isoform 2 [Homo
sapiens]
MCSQEAFQAQRSQLVELLVSGSLEGFESVLDWLLSWEVLSWEDYEGFHLLGQPLSHLARRLLDTVWNKGT
WACQKLIAAAQEAQADSQSPKLHGCWDPHSLHPARDLQSHRPAIVRRLHSHVENMLDLAWERGFVSQYECDEIRLPIFTPSQRARRLLDLATVKANGLAA
FLLQHVQELPVPLALPLEAATCKKYMAKLRTTVSAQSRFLSTYDGAETLCLEDIYTENVLEVWADVGMAGPPQKSPATLGLEELFSTPGHLNDDADTVLV
VGEAGSGKSTLLQRLHLLWAAGQDFQEFLFVFPFSCRQLQCMAKPLSVRTLLFEHCCWPDVGQEDIFQLLLDHPDRVLLTFDGFDEFKFRFTDRERHCSP
TDPTSVQTLLFNLLQGNLLKNARKVVTSRPAAVSAFLRKYIRTEFNLKGFSEQGIELYLRKRHHEPGVADRLIRLLQETSALHGLCHLPVFSWMVSKCHQ
ELLLQEGGSPKTTTDMYLLILQHFLLHATPPDSASQGLGPSLLRGRLPTLLHLGRLALWGLGMCCYVFSAQQLQAAQVSPDDISLGFLVRAKGVVPGSTA
PLEFLHITFQCFFAAFYLALSADVPPALLRHLFNCGRPGNSPMARLLPTMCIQASEGKDSSVAALLQKAEPHNLQITAAFLAGLLSREHWGLLAECQTSE
KALLRRQACARWCLARSLRKHFHSIPPAAPGEAKSVHAMPGFIWLIRSLYEMQEERLARKAARGLNVGHLKLTFCSVGPTECAALAFVLQHLRRPVALQL
DYNSVGDIGVEQLLPCLGVCKALYLRDNNISDRGICKLIECALHCEQLQKLALFNNKLTDGCAHSMAKLLACRQNFLALRLGNNYITAAGAQVLAEGLRG
NTSLQFLGFWGNRVGDEGAQALAEALGDHQSLRWLSLVGNNIGSVGAQALALMLAKNVMLEELCLEENHLQDEGVCSLAEGLKKNSSLKILKLSNNCITY
LGAEALLQALERNDTILEVWLRGNTFSLEEVDKLGCRDTRLLL

b

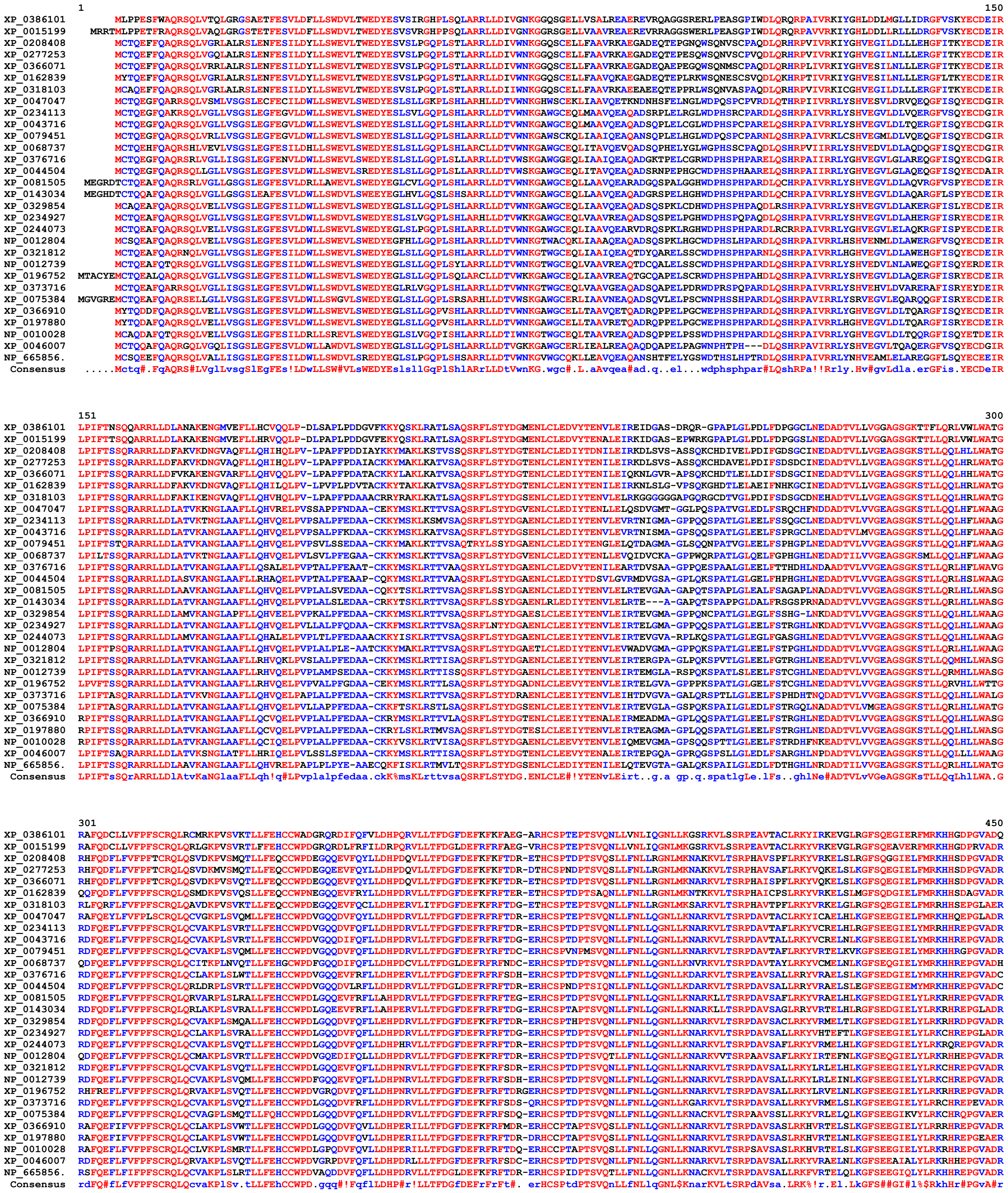
1
150
XP_0386101
XP_0015199
XP_0208408
XP_0277253
XP_0366071
XP_0162839
XP_0318103
XP_0047047
XP_0234113
XP_0043716
XP_0079451
XP_0068737
XP_0376716
XP_0044504
XP_0081505
XP_0143034
XP_0329854
XP_0234927
XP_0244073
NP_0012804
XP_0321812
NP_0012739
XP_0196752
XP_0373716
XP_0075384
XP_0366910
XP_0197880
NP_0010028
XP_0046007
NP_665856.
Consensus
151
300
301
450

```
            451                                                                                                                                                  600
XP_0386101  IVRLVRATPALRGLCHVPVFSWIVSRCHVELLQRWQGSSGGSLKTMTDMYFLIVRHLVLRSPLEGNRGSAEG--------VWRGRLPALLRLGELALGGLGAGCYVFSDGQLQAVGVSTEDLSLGFLVPSKSSAGAA---PQAHFEFLHV
XP_0015199  IVRLVGATPALRGLCHVPVFSWIVSRCHVELLRLRQGGGGGAPKTMTDMYFLIVRHLVLRSPLEGDRGPAGG--------VWRGRLPALLRLGELALGGLGAGCYVFSAGQLQAAGVSAEDLSLGFLVPSKGSAGGAGGSPEARFEFLHV
XP_0208408  IVCLVKATSALHGLCHVPVFSWIVSKCHKELLLH----GCSSLKTTTDMYLLILQHFVLHASPEDEGWHPGTTT-------LRPRLPTILHLGRLALWGLGTCSYVFSAKQLWASGMDEEDLSLGFLVRSKSF-SQE---SSAPMEFLHI
XP_0277253  IVHLVKATSALHGLCHVPVFSWIVSKCHKELLLH----GCGSLKTTTDMYLLILQHFVLHASPDDGGWHPRTTT-------LQPRLPTILHLGRLALWGLGTCNYVFSAKQLWASGVDEEDLSLGFLVRSKSF-SQE---SSAPMEFLHI
XP_0366071  IVHLVRAASALHGLCHMPVFSWIVSKCHKELLLH----GCGSLKTTTDMYLLILQHFVLHASPEDGGWRPGTSS-------LRPWLPTILHLGRLALWGLGTCSYVFSAKQLWESGVEEKDLSLGFLVPSKSF-SQE---SSAPVEFLHI
XP_0162839  LVQLVKATSALHGLCHVPVFSWIVSRCHKELLLH----GSGFLKTTTDMYLLVLRHFISHASPEDVAWGLGAST-------LRPRLPTILRLGRLALWGLGTCSYVFSAEQLRASGVDEEDLSLGFLVPSKSF-PPE---SSAPVEFLHI
XP_0318103  IVRLLKATSALHGLCHVPVFSWIVSRCHKELLQR----GCGSPKTTTDMYLLILRHFVMHNSPGAAAWGPGAAAWGPGVPCLRPWLPTILRLGRLALWGLGTCSYAFSAKQLWARGVEEEDLSLGFLVRSKSF-SQE---SAPSLEFLHI
XP_0047047  LVHLLKTTSALHGLCRLPVFSWIVSKCHQELLLL----GGGSLRTTTDMYLLILQHFLRHASPPDSAALSQDPS------LLRDRLPTLLHLGHLALWGLGTCCYVFSATQLQAAQVGTEDLSLGFLVRAKSI-VPG---SSPSLEFLHI
XP_0234113  LICLLKATSALHGLCYLPVFSWIVSRCHQELLLQ----GGGSLKTTTDMYLLILQHFLLHTSPPDTDPRGPGPS------LLRGRLPTLLRLGHLALWGLGTCCYVFSAKQLQAAQVDTDDIALGFLVRAKSV-VLG---SSAPLEFLHI
XP_0043716  LVCLLKATSALHGLCHLPVFSWIVSKCHQELLLQ----GGGSFKTTTDMYLLILQHFLLHAAPPDTDRRGLGPS------LLRGRLPTLLHLGHLALWGLGTCCYVFSAKQLQAAQVDTDDISLGFLVRAKSV-VLG---SSAPLEFLHI
XP_0079451  LVHLLKATSALHGLCHLPVFSWIVSKCHQELLLQ----GGGSLKTSTDMYLLILQHFLLHSSPPDTTRSSPGPS------LLSGRLTTLLHLGCLALWGLGMCCYVFSAKQLQAAQVDTEDISLGFLVRAKST-VPG---SSAPLEFLHI
XP_0068737  LVHLLRTTSALHGLCHLPVFSWIVSKCHQELLLQ----GGGSLKTSTDMYLLTLQHFLLRTSPPDLVPRSWGPS------LLRDRLPTLVHLGRLALWGLGLCCYVFSATQLQAAQVDSEDIALGFLVRAKSI-RPR---GSAPLEFLHI
XP_0376716  LLRLLRATSTLHGLCHLPVFSWIVSKCHRELLQH----GGGSLKTTTDMYLLILQHFLLHASPPDTASHGLGPS------FLRGRLPTLLRLGCLALRGLGTCCYVFSAKQLQAAQVDPEDVSLGFLVPVKSQGVSG---ASAPLEFLHI
XP_0044504  LLRLLRATSALRGLCRLPVFSWMVSKCHRELLQP----GGGSPKTTTDMYLLILQHFLLHSSSPDQAAHGPGPG------FLRGRLPSLLHLGCLALRGLGTCCYVFSAKQLQAAQVGPEDISLGFLVPAKGQGVRG---GSAPLEFLHV
XP_0081505  LVRLLRATSALHGLCHLPVFAWMVSKCHQELLLQ----GGGSPRTTTDMYLLILQHFLLHASPPDAAPHGPGPG------LLRGKLPTLLHLGRLALWGLGVSCYVFSATQLQAAHVDGADISLGFLVHAKSV-APG---GPPPLEFLHI
XP_0143034  LVRLLRATSALHGLCHLPVFAWMVSKCHQELLLQ----DGGSPRTTTDMYLLILQHFLLHASPPDAAPHGPGPG------LLRGRLPTLLHLGRLALWGLGVSCYVFSAQQLQAAHVDGADVSLGFLVHAKSA-APG---SPPPLEFLHI
XP_0329854  LISLLKATSALHGLCHLPVFSWMVSKCHQELLLQ----GGGSPTTTTDMYLLILQHFLQHTSPPDSTPPHLGAG------LLRGRLPTLLHLGRLALWGLGMCCYVFSAKQLQAAHVDSEDISLGFLVRAQRA-VSE---STAPLEFLHI
XP_0234927  LISLLRATSALHGLCHLPVFSWMVSKCHQELLLQ----GGGSPKTTTDMYLLILQHFLLHASPPDSAPGGLEPG------LLRGRLPTLLHLGQLALWGLGTCCYVFSAKQLQAAHVDSEDISLGFLVRAKRA-VPG---SAPPMEFLHI
XP_0244073  LIRLLKETSTLHGLCHLPVFAWMVSKCHQELLLQ----GGGPPKTTTDMYLLFLQHFLLHASPPDSARHTLGPS------LLRGRLPTLLHLGRLALWGLGMSCYVFSAQQLQAAQVNEEDISLGFLVHAQGV-VPG---STPRLEFLHI
NP_0012804  LIRLLQETSALHGLCHLPVFSWMVSKCHQELLLQ----EGGSPKTTTDMYLLILQHFLLHATPPDSASQGLGPS------LLRGRLPTLLHLGRLALWGLGMCCYVFSAQQLQAAQVSPDDISLGFLVRAKGV-VPG---STAPLEFLHI
XP_0321812  LVRLLKATSPLHGLCHLPVVSWMVSKCHQELLLH----GGGSPKTTTDLYLLILQHFLLHASPPDSVPCGLAPG------LLQGRLPSLLHLGQLAVWGLGTCCYVFSDKQLQAAHIDSEDISLGFLVHAKSV-VHG---GTAPLEFLHI
NP_0012739  LIRLLKTTSALHGLCHLPVVSWMVSKCHQELLLH----GGGSPKTSTDMYLLILQHFLLHASPPDSVPHSLGSH------LLRGRLPTLLHLGWLALWGLGMCCYVFSAKQLQAAHIDDEDISLGFLVHAKTV-GPG---STTPLEFLHI
XP_0196752  LIRMLRATSALHGLCHLPVITWMVSKCHQELLLP----GGGSPRTTTGMYLLIVQHFLLRASPPDAAPRSPGPG------LLRGRLPALLHLGWLALWGLGRCCYVFSSRQLQAAHVDGDDVSLGFLVRAKSV-APG---SVAPLEFLHI
XP_0373716  LISLLKATSALHGLCHLPVFSWMVSKCHQELLLQ----GGESLKTTTDMYLLILQHFLQHASPLDTAAQGLEPR------LLRARLPTLLHLGQLALQGLGTCCYVFSAQQLQAAHVDMEDIALGFLVRAGRA-LPG---STGPLEFLHI
XP_0075384  LIGLLRATSALHGLCHLPVFSWMVSKCHQELLLQ----GGGSPKTTTDMYLLILQHFLLHACQPDLVAHSVGPC------LLKARLATLLHLGRLALWGLGHCCYVFSAGQLQAAHVDAEDIALGFLVRARRV-VPG---SLAPLEFLHI
XP_0366910  LIRLLRATSALHSLCHLPVFSWMVSKCHQELLLQ----GGGSPKTTTDMYLLILQHFLLHASPPDSAAHGLGAG------LLRGRFPTLLRLGRLALWGLGTCCYVFSTKQLQAAHVDSEDVSLGFLVRAKRV-VPG---STAPLEFLHV
XP_0197880  LICCLRATSALHSLCHLPVFSWMVSKCHRELLLQ----GGVSPKTTTDMYLLILQHFLLHASPPDSAAHGLGAG------LLRGSLLTLLHLGRLALWGLGTCCYVFSPRQLQAAHVDSEEVSLGFLVRAKTV-VPG---STAPLEFLHI
NP_0010028  LLCLLRATSALHGLCHLPVFSWMVSKCHEELLLQ----GRGSPKTTTDMYLLILRHFLLHASPLPLATHGLGPS------LIQGRLPTLLHLGRLALWGLGTCCYVFSAKQLQAAHVDSEDLSLGFLVLAKRV-VPG---STAPLEFLHI
XP_0046007  LISLLQATPTLHGLCHLPVFSWMVSKCHQELLLQ----GGGALKTTTDMYLLILQHFLLHASPVATAAPRLAPC------PLRDWLPTLLHLGRLALWGLGTCCYVFTDKQLREASVEAADMTLGFLVHAQRA-GPS---GGASLEFLHI
NP_665856.  LIQLIQATSALHGLCHLPVFSWMVSRCHRELLLQ----NRGFPTTSTDMYLLILQHFLLHASPPDSSPLGLGPG------LLQSRLSTLLHLGHLALRGLAMSCYVFSAQQLQAAQVDSDDISLGFLVRAQSS-VPG---SKAPLEFLHI
 Consensus  l!.Ll.aTsaLhGLCHlPVFsWmVSkCHqELLlq....ggGspkTtTDMYlL!lqHfllhasppd.a....gp.      llrgrLptlLhLG.LALwGLGtccYVFSa.QLqAa.Vd.eD.sLGFLVraks...pg   s.aplEFLH!

            601                                                                                                                                                  750
XP_0386101  TFQCFLAALYLLLHSDVTPAAVGHLFH-RPRKCRVLDCLYPQLCIPAARREQG-----RPGPPLWGMETLDVQLTASFLAGLLSGANFSPLV---EGCGLEALLRKRASARKCLAQGIKRHFGSIPPAVPGELKSLHALPAFLWLIKSLY
XP_0015199  TFQCFLAALYLLLHGDVAPAAVGHLFH-RPRKSRVLELLYPRLCIPAARREEG-----RPGSLPWGAETVDVQLTASFLAGLLSGTNFAPLA---ESHGSEALLRKRASARKCLARGIERHFRSIPPAVPGELKSLHALPAFLWLIKSLY
XP_0208408  TFQCFFAAFYLLLGAEVTPSVIRCLFNHHHKKPSRSLAWLFPMSCVQSPEGEEE-----SVTALLQKAEPHNIQITAIFLAGLLSDEHWGLLA--ECQEPSTPLLQKQSRVRWCLARGLRRHFQSIPPAVPGEVKSMHAMPEFVWLIRSLY
XP_0277253  TFQCFFAAFYLLLGADVTPSVIRCLFNHHHKKPSRSLAWLFPMSCVQSPEGEEE-----SVTALLQKAEPHNIQITAVFLAGLLSHKHWGLLA--ECQEASTPLLQKQSHVRWCLARGLKRHFQSIPPAVPGEVKSMHAMPEFVWLIRSLY
XP_0366071  TLQCFFAAFYLLLDADVTPSVIRHLFNHHHKKPSRPLAWLFPVSCIQSPEREEA-----SVAELLQKAEPHNIQITAVFLAGLLSQEHWGLLA--ECQGASTPLLQKQNLTRWCLARGLRRHFQSIPPAVPGEVKSMHAMPEFVWLIKSLY
XP_0162839  TLQCFFAAFYLLLGADVNPSAIRCVFGGHKKPHQPLPRLFPMACFQSPEPEEE-----SVLTLLQRAETHNVRITAVFLAGLLSQEHWRLVT--ECRGTSTPLLQKKSRVRRCLARGLRRHFRSIPRAVPGEVKSMHAMPEFVWLIRSLY
XP_0318103  TLQCFFAAFYLLLGADVRASALRHLFHRREQPGRSLARRFPVSCLRAPEPAEAEEAAASVSGLLQKAEPHNVQITAAFLAGLLSREHRGLVA--PGQGAPGPLPRKQSLVRRSLALALRRHFRAIPPAVPGEAKSMHAMPEFVWLIRSLY
XP_0047047  TFQCFFAAFYLVLSPDFPPTMLRHLFNCKGPGGSKLARLLPILCIQSTECKEN-----GVVTLLQKAEPHNLQIMAAFLAGLLSREHWGLLA--ECQLSQKALLQRHTSARCCLARSLRRHFHSIPPAVPGEAKSMHAMPSFLWLIRSLY
XP_0234113  TFQCFFAAFYLVLSSDLPPTTLRHLFNCCRTGGSLLARLLPALCIQRSESKES-----SVVAWLQKAEPHNLQITAAFLAGLLSREHRGLLA--ECQASEKALLQRHTCARWCLARSLRKYFHSIPPGVPGEAKSMHAMPGFLWLIRSLY
XP_0043716  TFQCFFAAFYLVLSSDLPPTTLRHLFSCCRSGSSPLARLLPALCVQHSERKED-----SVAAWLQKAEPHNLQITAAFLAGLLSREHRGLLT--ECQVSKKALLRRHACARWCLARSLRKHFHSIPPAVRGEAKSMHALPGFLWLIRSLY
XP_0079451  TFQCFFAAFYLVLSADLPPTILRHLFNCQRPGGSLLARLLPMLCVPGSEHKEG-----TVVSLLQEAEPHNLQITAAFVAGLLSQEHRGLLV--ECQASKKALLRRHACARWCLAHSLRKHFHSIPPAVPGEAKSMHAMPGFLWLIRSLY
XP_0068737  TFQCFFAALYLILSADLPPTTLRYLFNCQSLGGFLKARLLPTLCIQSSECKED-----NVVALLQKAEPHNLQTTASFLAGLLSQEHRGLLA--QCQASEKALLRRHACVRWCLARSIRKHFHSIPPAVPGEAKSMHAMPGFLWLIRSLY
XP_0376716  TFQCFFAAFYLVLSADMPPTSLRHLFDCRGPGSSPLARLLPMLCIQGSECKEG-----GLAALLQKAEPHNLQITAAFLAGLLSREHRGLLA--ECQASGKALLRRHACARWCLARSLRQHFHSIPSAVPGEAKSLHAMPRFLWLIQSLY
XP_0044504  TFQCFFAAFYLVLSADMPPTSLRHLFGCRRPSSSPLARLLPTLCIQRSEGPEG-----GVAAWLREAEPHNLQITAAFVAGLLSREHRSLLA--ECQASGKALLRRHACARWYLARSLRQHFHSIPPAVPGEAKSVHALPGFLWLIRSLY
XP_0081505  TFQCFFAAFYLVLSADVRPSLLRHLFNCRPTGSSPLARLLPALCVQGPPSEEG-----SVEAWLQQAEPHNLQITAAFLAGLLSREHRGLLA--ECQASEKALLRRQACARRCLARSLRKHFCSIPPALPGEAKSMHALPGFLWLVRSLY
XP_0143034  TFQCFFAAFYLVLSADVRPSSLRHLFNCRPTGSSPLARLLPALCVQGPPREEG-----GVEAWLQQAEPHNLQITAAFLAGLLSREHRGLLA--ECQASEKALLRRQACARRCLARSLRKHFCSIPPALPGEAKSMHALPGFLWLVRSLY
XP_0329854  TFQCFFAALYLVLSADMPPSSLRYLFNCHPPESSLLARLLPALCVPGSRCKED-----SVAVLLQEAEPHNLQITAAFLAGLLSQEHRSLLA--ECQASEKALLRRQDCARRCLAHSLHKHFQSIPPAVPGEAKSMHAMPGFVWLIRSLY
XP_0234927  TFQCFFAAFYLVLSADVPPSSLRYLFSCGRPGSSLLARLLPAMCVQGSRCKED-----SVEALLQEAEPHNLQITAAFLAGLLSREHRGLLA--KCQGSETALRQRQACARWCLARSLHKHFHSIPPAVAGEAKSMHAMPGFIWLIRSLY
XP_0244073  TFQCFFASLYLALSVNVTPSSLRHLFSCGGTGSSPLARLLPTLCVQGPRPREG-----SVEALLQEAEPHNLQITAAFLAGLLSQEHRGLLA--ECQASEKALLRRQACARWCLARSLQKHFHSIPPALPGEAKSMHAMPGFVWLIRSLY
NP_0012804  TFQCFFAAFYLALSADVPPALLRHLFNCGRPGNSPMARLLPTMCIQASEGKDS-----SVAALLQKAEPHNLQITAAFLAGLLSREHWGLLA--ECQTSEKALLRRQACARWCLARSLRKHFHSIPPAAPGEAKSVHAMPGFIWLIRSLY
XP_0321812  TFQCFFAALYLVLSADLSPSLLRQLFSCHGPRSSLLARLLPTMCVPRSEHQEG-----STAALLQEAEPHNLQITAAFLAGLLSREHQGLLA--GSQVSEKALLQRQACARSCLSCSLHKHFHSIPPAVPGEAKSMHAMPGFIWLIRSLY
NP_0012739  TFQCFFAALYLVLSTDVSPSLLRQLFICHGPRSSLLARLLPTTCVPRSERKEG-----SLAALLQEAEPHNLQITAAFLAGLLSREHRGLLA--ECQANEEALLRLQGRAQGCLSRSLHQHFRSIPPAVPGEAKSMHAMPGFLWLIRSLY
XP_0196752  TFQCFFAAFYLVLSADVPPSSLRRLFRCHTPGGSLLARLLPAACVRRPAREEG-----GVAALLQEAEPHNLQITAAFLAGLLSREHRGLLA--EGQASEKALLRRRVCARWCLSRSLHKHFHAIPPAVPGEAKSMHAMPGFVWLSRSLY
XP_0373716  TFQCFFAAFYLVLSADMPPSSLRHLFSCHRPGSSLLARLLPRMCVPGAGPKEG-----SVAALLQGVDPHNLQITAAFLAGLLSQEHCGLLA--ECQVSEKALLRRQACARGCLARSLHRHFHSIPPAVPGEAKSMHAMPGFVWLIRSLY
XP_0075384  TFQCFFAAFYLVLSTDAPPSLLRQVFSCRRPGSPLVARLLPALCVPGPGHREG-----SVAALLQEAEPHNLQITAAFVAGLLAQEHQGLLA--ECQASEKALLRRQACARWCLARSLQRHFHSIPPAVPGEAKSMHAMPGFLWLIRSLY
XP_0366910  TFQCFFAAFYLALSADTPPSSLRHLFNGHGPGCSPLARVLPKLCVRGSGCKEG-----SVAALLQGAELHNLQITAAFLAGLLSQERQGLLA--ECQVSEKALLCRQACTRQCLARSLRKHFRSIPPAVPGEAKSMHAMPSFIWLVRSLY
XP_0197880  TFQCFFAAFYLALSADTPPSSLRHLFNCHGPGCSPLAGVLSKLRMRDSGCKEG-----SVAALLQGAEPHNLQITAAFLAGLLSQEHRGLLA--ECQVFEKALLWRQACTRRCLARSLRKHFRSIPPAVPGEAKSVHAMPSFIWLIRSLY
NP_0010028  TFQCFFAAFYLALSADTPPSSLRHLFQDHRPESSPLARVLPKLFLRGSRCREG-----SVAALLQGAEPHNLQITGAFLAGLLSQEHRSLLA--ECQASETALLRRWDCVRRCLTRSLREHFRSIPPALPGEAKSMHALPGFLWLIRSLY
XP_0046007  TFQCFFAAFYLVLGADVPPSSLRHLFRCGRPGGWLRGRLLPALCVPGSGTREG-----SVAALLQEAETHNLQMTAAFLAGLLSREHRDLLAAAEGRASGKALLQRQACARRCLARSLHRHFRGIPAAVPGEAKSMHAMPNFVWLIRSLH
NP_665856.  TFQCFFAAFYLAVSADTSVASLKHLFSCGRLGSSLLGRLLPNLCIQGSRVKKG-----SEAALLQKAEPHNLQITAAFLAGLLSQQHRDLLA--ACQVSERVLLQRQARARSCLAHSLREHFHSIPPAVPGETKSMHAMPGFIWLIRSLY
 Consensus  TfQCFfAAfYL.LsaDvpPs.lrhLF.c..pgss.larllP.lC.......#g     svaalLq.AEph#lQiTAaFlAGLLS.ehrglLa  ecq.sekaLLrrqacaR.CLarslr.HF.sIPpAvPGEaKS$HA$PgF.WL!rSLY

            751                                                                                                                                                  900
XP_0386101  EMQDEGLARRALRGFKVEHVKLTYCGVGPAECAALAFVLRFLRHPVSLQLDYNSVGDVGLEQLLPCLKVCRALYLRDNNISDHGICKLVDQALLCDSFQKLALFHNKLTDDSAHSLAKLLKYKENFLALRLGNNHITAVGAKVLAEGLER
XP_0015199  EMQDEGLARRAVRGFEVEHVKLTYCGVGPAECAALAFVLRFLRRPVSLQLDYNSVGDVGLEQLLPCLKVCRALYLRDNNISDHGISKLVDQALLCDSLQKLALFNNKLTDDSAHSLAKLLKYKENFLALRLGNNHITAVGAKVLAEGLEG
XP_0208408  EMQDEHLAREAVCKLEVEHLKVTYCNIGPAECAALAFVLKNLKQPVALQLDYNSVGDVGVEQLLPCLRVCQALYLRDNNISDQGICRLVDQALQCDQFQKLALFNNKLTDNCAHSLASLLKYKQNFLALRLGNNHITAVGAKVLAEGLKD
XP_0277253  EMQDEHLAREAVCKLEVGHLKVTYCNIGPAECAALAFVLKNLKQPVALQLDYNSVGDIGVEQLLPCLRICQALYLRDNNISDQGICRLVDQALQCDQFQKLALFNNKLTDDCAHSLASLLKHKQNFLALRLGNNHITAVGAKVLAEGLKD
XP_0366071  EMQDERLAREAVCKLEVEHLKVTYCNIGPAECAALAFVLKNLKQPVALQLDYNSVGDIGVEQLLPCLHVCQALYLRDNNISDQGICTLVDQVLQCDHFQKLALFNNKLTDDCAHSLASLLKNKQNFLALRLGNNHITAVGAKVLAEGLKH
XP_0162839  EMQDEHLARKAVSKLEVGHLKVTYCNIGPAECAALAFVLKNLQQPVALQLDYNSVGDIGVEQLLPCLSVCRALYLRDNNISDQGICRLVDQALQCDQFQKLALFNNKLTDDCAHSLASLLKYKQNFLALRLGNNHITAVGAKVLAEGLKD
XP_0318103  EMQDERLAREAVSKLGVGHLKVTYCNIGPAECAALAFVLKNLQRPVALQLDYNAVGDAGLEQLLPCLPVCQALYLRDNNISDLGICRLVDQALQCEQFQKLALFNNKLTDDCAHSLATLLKYKQNFLALRLGNNHFTAVGAKVLAEGLKG
XP_0047047  EMQEERLAQEAVHRLDIEHLKLTFCSVGPTECAALAFVLRHLRRPVALQLDHNSVGDIGVEQLLPCLRVCKALYLRDNDISDRGVCKLIEHALHCEQLQKLALFNNRLTDGCAHSIAKLLACKHNFLALRLGNNRITAVGAKVLAQGLRD
XP_0234113  EMQEERLAREAVRGLDVEHLKLTFCGVGPAECAALAFALQHLRQPVALQLDYNSVGDIGVEQLLPCLGVCKALYLRDNNISDRGICKLIEHALHFEQLQKLALFNNKLTDGCARSMAELLARKQNFLALRLGNNHITAVGAEVLAQGLRD
XP_0043716  EMQEEQLAQEAVRGLEVEHLKLTFCGVGPAECAALAFALQHLRRPVALQLDHNSVGDIGVEQLLPCLSVCKALYLRDNNISDRGICKLIEHGLHCEQLQKLALFNNKLTDGCARSVAELLACKQNFLALRLGNNRITAAGAEVLAQGLRD
XP_0079451  EMQEERLAREAACGLDVEHLKLTFCSVGPTECAALAFVLRHLRRPVALQLDYNSVGDVGVEQLLPCLGVCKALYLRDNNISDRGICKLIERALHCEQLQKLALFNNKLTDGCAHAMAQLLACKQNFLALRLGNNHITASGAEVLAQGLQD
XP_0068737  EMQEERLAQEAVRGLDVEHLKLTFCNVGPTECAALAFVLRHLRRPVALQLDHNSVGDTGLEQLLPCLGVCKALYLRDNNISDQGISKLIEQALHCEQLQKLALFNNKLTDGCAHSIAKLLACKHNFLALRLGNNHITAVGAEVLAQGLRN
XP_0376716  EMQEERLAREAVRGLSVGHLKLTFCGVGPIECAALAFVLRHLRRPVALQLDHNSVGDIGVEQLLPCLGVCKALYLRDNNISDRGICKLIEHALPCEQLQKLALFNNKLTDDCAHSMAKLLACKRNFLALRLGNNRISAVGAAVLAEGLRS
XP_0044504  EMQEEQLAREAVRGLSVGHLKLTFCGVGPAECAALAFVLRHLRRPVALQLDYNSVGDIGVEQLLPCLGVCKALYLRDNDISDLGICKLIEHALSCEQLQKLALFNNRLTDDCTHSMAKLLACKRNFLALRLGNNHITAVGAAVLAQGLRS
XP_0081505  ELREERLARKAVRGLDVGHLKLTFCSVGPAECAALAFVLRHLRRPVALQLDHNSVGDVGVEQLLPCLGVCKALYLRDNNLSDRGVCTLAEHALRCEELQKLALFNNKLTDGCAQALARLLECRQNFLALRLGNNRITAAGAQELARGLRA
XP_0143034  ELREERLARKAVRGLDVGHLKLTFCSVGPAECAALAFVLRHLRRPVALQLDHNSVGDVGVEQLLPCLGVCKALYLRDNNISDRGMCTLAEHALRCEELQKLALFNNKLTDGCAQALARLLECRQNFLALRLGNNHITEAGAQVLARGLRA
XP_0329854  EMQEERLAREAVRRLNVEHLKLTFCSVGPAECAALAFVLQHLRWPVALQLDHNSVGDIGVEQLLPCLGVCKALYLRDNNISDRGICKLIEHALHCEQLQKLALFNNKLTDGCAHSMARLLECRRNFLALRLGNNHITAVGAQVLAQGLRA
XP_0234927  EMQEERLAQEAVRGLDVGHLKLTFCSVGPAECAAMAFVLRHLRRPVALQLDHNSVGDVGVEQLLPCLSVCTALYLRDNNISDRGICQLIERALHCEQLQKLALFNNKLTDGCAHSMARLLMCKQNFLALRLGNNHITAAGAVVLAQGLRA
XP_0244073  ELREERLAREAVHGLRVEHLKLTFCGVGPTECAALAFVLQHLRRPVALQLDHNSVGDVGVEQLLPCLHVCKALYLRDNNISDRGICKLLEHALHCEALQKLALFNNKLTDGCAQSVARLLECRQNFLALRLGNNHITSVGAQVLARGLRA
NP_0012804  EMQEERLARKAARGLNVGHLKLTFCSVGPTECAALAFVLQHLRRPVALQLDYNSVGDIGVEQLLPCLGVCKALYLRDNNISDRGICKLIECALHCEQLQKLALFNNKLTDGCAHSMAKLLACRQNFLALRLGNNYITAAGAQVLAEGLRG
XP_0321812  EMQEERLAREAVRGLKVGHLKLTFCGVGPAECAALAFVLRHLRRPVALQLDHNSVGDVGVEQLLPCLNVCKALYLRDNNISDRGVCKLVERALHCEQLQKLALFNNKLTDGCAPSMARLLSCKRNFLALRLGNNHITATGARVLAEGLRA
NP_0012739  EMQEERLAREAVRGLTVGHLKLTFCGVGPPECAALAFVLRHLRRPVALQLDHNSVGDIGVEQLLPCLSVCKALYLRDNNISDRGICKLIEHALHCEQLQKLALFNNKLTDGCAHSMARLLACKQNFLALRLGNNHITAAGAQALAEGLRA
XP_0196752  EMQEERLAREAVRGLKVGHLKLTFCGVGPAECAALAFVLRHLRRPVALQLDHNSVGDVGVEQLLPCLDVCKALYLRDNNISDRGICKLVEHALHCEQLQKLALFNNKLTDGCAHSVARLLACRQNFLALRLGNNHITASGAQVLAEGLRA
XP_0373716  EMQEERLAREAVRGLDVGHIKLNFCSVGPTECAALAFVLRHLRRPVALQLDHNSVGDIGVQQLLPCLGVCKALYLRDNNISDRGICKLVERALHCEQLQKLALFNNRLTDGCAHAMARLLSCKQNFVALRLGNNHITASGAEVLAQGLRA
XP_0075384  ELQEERLAREAVRGLDVGHLKLTFCGVGPVECAALAFVLRHLRRPVALQLDHNSVGDIGVEQLLPCLRVCKALYLRNNNISDRGVCKLVEHALHCEQLQKLALFNNKLTDGCTHAMARLLTCRQNFLSLRLGNNNITAAGAEVLAGGLRA
XP_0366910  EMQEERLAWEAVRRLDVGHLKLTFCSVGPAECAALAFVLRHLRQPVALQLDHNSVGDIGVEQLLPCLGVCKALYLRDNNVSDRGICKLTEHALRCERLQKLALFNNKLTDGCAHSMAKLLACKQNFLALRLGNNHITAAGAEVLAQGLRA
XP_0197880  EMQEERLAREAVRRLDVGHLKLTFCSVGPAECAALAFVLRHLRQPVALQLDHNSVGDMGVEQLLPCLGVCKALYLRDNNVSDRGICKLTEHAVRCEQLQKLALFNNKLTDGCAHSMAKLLACKQNFLALRLGNNHITAAGAEVLAQGLRA
NP_0010028  EMQEERLAREAVCRLNVGHLKLTFCGVGPAECAALAFVLRHLRRPVALQLDHNSVGDIGVEQLLPCLGVCKALYLRDNNISDRGICKLVEHALRCEQLQKLALFNNKLTDGCAHSMARLLACKQNFLALRLGNNHITAAGAEVLAQGLRT
XP_0046007  EMQEERLAREAVRGLDVGHLKLTFCGMGPAECAALAFVLRHLRQPVALQLDHNSVGDIGVEQLLPCLGVCKALYLRNNNISDRGICLLVEHALRCEPLQKLALFNNRLTDGSARALARLLACKRNFLSLRLGNNHFTAAGAQVLAEGLRG
NP_665856.  EMQEEQLAQEAVRRLDIGHLKLTFCRVGPAECAALAFVLQHLQRPVALQLDYNSVGDVGVEQLRPCLGVCTALYLRDNNISDRGARTLVECALRCEQLQKLALFNNKLTDACACSMAKLLAHKQNFLSLRVGNNHITAAGAEVLAQGLKS
 Consensus  E$Q#ErLAreAvrgl.VgHlKlT%C.!GPaECAALAFVLrhLrrPVaLQLDhNSVGDiGvEQLLPCL.VCkALYLRDNNISDrG!ckLv#.AL.C#qlQKLALFNNkLTDgcAhs.A.LL.ckqNFLaLRLGNNhITA.GA.VLA.GLr.
```

```
           901                                                                                                                                       1043
XP_0386101 NHSLQFLGLWGNAVGDEGAQALADALHGHRSLKWLSLVGNDIGSVGAQALALMLGKNVVLEELCLEENRFSDQDVCSLAEGLKKNSTLKVLKLSNNNITCQGVVSLLQTLKKNDTLKSIWLRGNTFTLEEIESLSYMDPRLLL
XP_0015199 NHSLQFLGLWGNTVGEEGAQALADALHGHRSLKWLSLVGNNVGSVGARALALMLGKNVVLEELCLEENRLNDQDVCSLAEGLKKNSSLKVLKLSNNNITYQGVVSLLQTLKKNDTLKSIWLRGNTFTPEEIESLSFMDPRLLL
XP_0208408 NNSLQFLGLWGNAVGDEGAQALASALHDHRSLKWLSLVGNNIGSLGAQALALMLEKNVSLEELCLEENHLQDEDMCTLVHGLKKNSSLKVLKLSNNSITQQGVSALLQVLQSNTSIKSVWLRGNTFTPEEIEQLSLMDARLLL
XP_0277253 NNSLQFLGLWGNAVGDEGAQALASALHDHRSLKWLSLVGNNIGSLGAQALALMLEKNVSLEELCLEENHLQDEDMCTLVHGLKKNSSLKVLKLSNNSISQQGVSALLQVLQSNTSIKSVWLRGNTFTPEEIKQLSLMDTRLLL
XP_0366071 NDSLQFLGLWGNAVGDEGAQALASALHDHRSLKWLSLVGNNIGSLGVQALALMLEKNVSLEDLCLEGNHLQDEDMCTLVHGLKKNSSLKVLRLSNNGITHKGVSALLQVLQSNSSIKSIWLRGNTFTPEEIEQLSLVDARLLL
XP_0162839 NDSLQFLGLWGNTVGDEGAQALACALHDHHSLKWLSLVGNNIGSVGTQALALMLEKNVSLEELCLEENHLQDEDMCTLVDGLKKNSSLEVLKLSNNRITHRGVSALLQVLESNATIRSIWLRGNTFTPEEIEQLSLMDSRLLF
XP_0318103 NDSLQFLGLWGNKVGDEGARALASALHDHPSLKWLSLVGNNIGSLGAQALALMLEKNVSLEELCLEENHLQDEDMCVLVRGLKKNSSLKVLKLSNNSITLQGVSVLLQVLEGNATIKSIWLRGNIFTTEEIEQLSIMDARLLL
XP_0047047 NSSLQFLGFWGNTVGDKGAQALAEALSDHQSLKWLSLVGNNIGSVGAQALALMLEKNVVLEELCLEENHLHNEGVCSLAEGLTRNSSLKVLKLSNNHITYVGAEVLLRALEKNDAILEVWLRGNAFSPEEIQILSHKDIRLLL
XP_0234113 NCSLQFLGFWGNRVGDKGAQALAEALSDHQSLKWLSLVGNSIGSVGAQALARMLEKNMVLEELCLEENHLHDEGVCFLAEGLKRNSSLKVLKLSNNSITYAGAEALLQTLERNNTILEVWLRGNTFSPEEIETLSHKDTRLLL
XP_0043716 NVSLQFLGFWGNRVGDKGAQALAEALSNHRSLKWLSLVGNDIGSLGAQALALMLERNVVLEELCLEENHLHDEGVCFLAEGLKRNSSLKVLKLSNNCISCVGAEALLQALERNDTILEVWLRGNTFSPEEIETLSHKDTRLLL
XP_0079451 NTSLQFLGFWGNKVGDKGAQALAEALSDHQSLKWLSLVGNNIGSVGAKALALMLEKNVVLEELCLEENHLHDEGVCSLAEGLKRNSSLKVLKLSDNFITCAGAEALLQALGRNDTILEVWLRGNTLSPEEMETLSRKDSRLLL
XP_0068737 NNSLQFMGFWGNKVGDKGAQALAEALSDHQSLKWLSLVGNNIGSVGAQALALMLEKNMVLEELCLEENYLHDEGVCFLAEGLQRNSSLKILKLSNNFITCTGAEALLWALERNNTIREIWLRGNTFSPEEIKMFSHKDTRLLL
XP_0376716 NSSLQFLGFWGNRVGDEGAQALAEALGSHQSLKWLSLVGNNIGSMGAQALALMLERNVVLEELCLEENHLQDEGVGSLAEGLKRNSSLKVLKLSNNCITYLGAEALLQALERNNTILEVWLRGNAFSPEEAEELSHRDTRLLL
XP_0044504 NSSLQFLGFWGNRVGDQGAQALAEVLGDHQSLKWLSLVGNNIGSVGAQALALMLERNMVLEELCLEENHLQDEGVCSLAKALKRNSSLKVLKLSNNRVTYLGAEALLQALERNDTILEVWLRGNTFSPEEIEKLSHRDSRLLL
XP_0081505 NSSLQFLGFWGNEVGDKGAQALAEALGDHQSLRWLSLVGNRIGSVGAQALASMLEKNVALEELCLEENQLGDEGVRSLAEGLRRNSSLKVLKLSNNRVTYRGAEALLQALERNDTVREVWLRGNAFSPEEMETLGRRDARLLL
XP_0143034 NTSLQFLGFWGNEVGDKGAQALAEALGDHQSLRWLSLVGNRIGSVGARALASMLEKNMALEELCLEENQLGDEGVCSLAEGLRRNPSLKVLKLSNNCVTYRGAEALLRALERNDTVREVWLRGNAFSPEEMETLGHRDARLLL
XP_0329854 NASLQFLGFWGNKVGDKGAQALAEALGDHQSLRWLSLVGNNIGSVGAQALALMLEKNVALEELCLEENHLQDEGVCSLAEGLKRNSSLKVLKLSNNCVTYLGAEALLQALARNDTILEVWLRGNTFSPEEIEKLSHRDTRLLL
XP_0234927 NTSLQFLGFWGNKVGDKGAQALAEALGNHPSLKWLSLVGNSIGSAGAQALAVMLEKNVALEELCLEENHLQDEGVCSLAEGLQRNSSLKVLKLSNNCITYRGAEALLQTLERNDTILEVWLRGNTFSPEEIEKLSHRDARLLL
XP_0244073 NTSLQFLGFWGNKVGDKGAQALAEALGDHQSLRWLSLVGNDVGSAGAKALALMLEKNVALEELCLEENHLQDEGVCSLAEGLTRNSSLKVLKLSNNCVTYRGAQALLQALERNSTILEVWLRGNTFSPEEIEQLSHRDTRLLL
NP_0012804 NTSLQFLGFWGNRVGDEGAQALAEALGDHQSLRWLSLVGNNIGSVGAQALALMLAKNVMLEELCLEENHLQDEGVCSLAEGLKKNSSLKILKLSNNCITYLGAEALLQALERNDTILEVWLRGNTFSLEEVDKLGCRDTRLLL
XP_0321812 NASLQFLGLWGNKVGDEGAQALAEALGDHQSLRWLSLVGNNIGSTGAQALALMLEKNMALEELCLEENRLQDEGVCSLAEGLEKNSSLKVLKLSDNSITYFGAEAVLRALERNDTILEVWLRGNTFSAEEVERLGQRDTRLLL
NP_0012739 NTSLQFLGFWGNKVGDEGAQALAEALGDHQSLRWLSLVGNDIGSVGARALALMLEKNVALEELCLEENHLQDEGVCSLAKGLERNSSLKVLKLSNNCITYLGAEGLLQALEKNDTILEVWLRGNTFSLEEMERLSQKDTRLLL
XP_0196752 NASLQFLGFWGNKVGDEGAQALAEALGDHQSLKWLSLVGNNIGSVGAQALALMLEKNVTLEELCLEENHLRDEGVCSLAKGLQRNSSLKVLKLSNNHITDLGAEVLLQALERNDTILEVWLRGNTFSPEETERLSQRDTRLLL
XP_0373716 NASLQFLGFWGNKVGDKGAQALAEALGDHQSLRWLSLVGNSIGSVGAQSLALMLEKNVALEELCLEENHLGDEGVCSLARGLKRNSSLKVLKLSNNCVTCRGAEALLQALERNDAIREVWLRGNAFSPEDVEQLCRRDARLLL
XP_0075384 NASLQFLGFWGNKVGDKGAQALAEALGDHTNLKWLSLVGNNIGSVGAQALAVMLEKNAVLEELCLEENHLQDEGVCSLTDGLQRNASLKILKLSNNGITDLGAEALLQALERNNTIQEVWLRGNSFSPEEMEKLSHRDARLLL
XP_0366910 NTSLQFLGFWGNKVGDRGAQALAEALGDHQSLRWLSLVGNNIGSVGAQALALMLEKNVALEELCLEENHVQDEGVCCLAKGLEKNSSLKVLKLSNNRITSLGAEALLQALEKNDTILEVWLRGNTFSPEEIEKLSHRDTRLLL
XP_0197880 NASLQFLGLWGNKVGDRGAQALAEALGDHRSLRWLSLVGNDIGSVGAQALALMLEKNMALEELCLEENHVQDEGVCCLAKGLEKNSSLKVLKLSNNGITSLGAEALLQALEKNDTILEVWLRGNTFSPEETEKLSHRDTRLLL
NP_0010028 NNSLQFLGFWGNQVGDEGAQALAAALGDHQSLRWLSLVGNNIGSVGAQALALMLEKNMALEELCLEENHVQDEGVCFLAKGLARNSSLKVLKLSNNHISSLGAEALLRALEKNDTILEVWLRGNTFSPEEIEKLSHQDTRLLL
XP_0046007 NTSLQFLGFWGNKVGDHGAQALAEALGKHENLKWLSLVGNNIGRVGARALALMLEQTMSLEELCLEENQLEDEGVCSLAEGMKRNSSLKILKLSNNGVTHLGATALLQALEENATLQEVWLRGNAFSPEELERLSRQDTRLLL
NP_665856. NTSLKFLGFWGNSVGDKGTQALAEVVADHQNLKWLSLVGNNIGSMGAEALALMLEKNKSLEELCLEENHICDEGVYSLAEGLKRNSTLKFLKLSNNGITYRGAEALLQALSRNSAILEVWLRGNTFSLEEIQTLSSRDARLLL
 Consensus N.SLQFLGfWGN.VGD.GAQALAeALgdHqsLkWLSLVGNnIGSvGAqALAlMLekNv.LEELCLEENhlqD#gvcsLaeGLkrNSsLK!LKLSNN.!T..GaeaLLqaLerNdtile!WLRGNtFspEEi#.Ls..D.RLLL
```

Supplementary Figure S1. Amino acid sequences and alignment of NOD2 proteins from phylogenetically diverse mammals. (a) Amino acid sequences of NOD2 proteins in FASTA format. **(b)** Multiple sequence alignment of NOD2 amino acid sequences. Red fonts indicate identical or highly similar residues that are conserved in at least 90% of the species. Blue fonts indicate identical or highly similar residues that are conserved in more than 50% of the species.

```
         1                                                                                                100
   Human TCTGACTTAGGTGATGGAACAGAATACAGAAATAGAATAAAGGAAAAATTTTGCATCACTTGGGACAAGAAGTCTTTGGCTGGAAAGCCTGAAGATTTCC
     Dog TCTGACTTAGGTGATGGAACAGAATACAGGATCCAAATAAAGGAAAAATTTCGTATCATGTGGGATAAGAAGTGTTTGTTTGGGGGACCTGAAGATTTCC
Pangolin TCTGACTTGGGTGATGGAATGGAATACAGAATTCAAATAGAGAAGAGGGTTTGCATGCCATGGG-TAAGAAGGGTTTGCTTGGAGAACCTGAAGAGTTCC

         101                                                                                              200
   Human ATCATGGAATTGCAGAGAAAGATAGAAAACTGTTGGAACACTTGTTCGATGTGGATGTCAAAAACCGGTGCACAGCCACAGATCGTGGTGCTTCAGGGAGC
     Dog ATCCTGGAATTGCTCAGGAAGATCGAGAACTGTTGGAACATCTGTTTGATGTGGATGTGAAAAACCGGCGAGCAGCCACAGACAGTTGTGCTCCAGGGGGC
Pangolin TCTATAGATTTGCTCAGGAAGATGGAGAACTGTTGGAGCGTTTGTTTGATGTCGATGTCGGAACC--CGGAGAGCCCGAGGCAGTGGTGCTGCAGGGGGC

         201                                                                                              300
   Human TGCTGGAGTTGGGAAAACAACCTTGGTGAGAAAGGCAATGTTAGATTGGGCAGAGGGCAGTCTCTACCAGCAGAGGTTTAAGTATGTTTTTTATCTCAAT
     Dog TGCTGGAGTTGGGAAAACCAGCTTGGTGAGAAAAGCAATAGTAGATTGGGCTGAGGGCAATCTCTATCAGCAGAAGTTTAGCTATGTTTTTTATCTCAAT
Pangolin TGCTAGAGCTGGGAAAACGCCCTTGGTGAGAAAGGCATTGATGGATTGGGCACCGGGCAGTCTCTGTCAGCAGAGGTTTACTGAGGGTTTTTATCTCTGC

         301                                                                                              400
   Human GGGAGAGAAATTAACCAGCTGAAAGAGAGAAGCTTTGCTCAATTGATATCAAAGGACTGGCCCAGCACAGAAGGCCCCATTGAAGAAATCATGTACCAGC
     Dog GCAAGAGAAATCAACCAGTTGAGAGAACGGAGCTTTGTTCAAATGATATCCAAGGACTGGCCCAGCACAGAAGGCCCCATCGAAAGGATAATGACCCAGC
Pangolin ATGAGATAAATC------TCTGCTGAGAGCAGCTTGGTGCACATGATATCAAAGGACTGGCCCAGCACAGAAGGCAACATTGAAAGGATCACGTCCCCAC

         401                                                                                              500
   Human CAAGTAGCCTCTTGTTTATTATTGACAGTTTCGATGAACTGAACTTTGCCTTTGAAGAACCTGAGTTTGCACTGTGCGAAGACTGGA-CCCAAGAACACC
     Dog CGAGTAGTCTCCTTTTTATTATTGATAGTTTTGATGAACTGAACTTCGCCTTTGAGGAACCGGACTTTGTGCTATGTGAAGACTGGA-CCCAGGTACACC
Pangolin GGAGCAGTCTCCTTTTTATTATCGATAGTTTCGATGAACTGAACTTTGCATTTGAGGAACTGGAATTGATACTGTGTGAAAACTGGAACCCAGGTGTACC

         501                                                                                              600
   Human CAGTGTCCTTCCTCATGAGTAGTTTGCTGAGGAAAGTGATGCTCCCTGAGGCATCCTTATTGGTGACAACAAGACTCACAACTTCTAAGAGACTAAAGCA
     Dog CCGTGTCCTTCCTTATGAGTAGTCTGCTGAGGAAAGTAATGCTCCCTGAGTCCTTTTTATTGGTGACAACGAGACTCACGGCTTGTAAGAAGCTAAAGCC
Pangolin CAGTGTCGTTCCTCATGAGGAGCTGGCTGAGGCACATGGTGCTCC--GAGTTGTTCTTATTGGTGACCACAAGGCTCACAGCTTGTAAGAAACTAAAGCC

         601                                                                                              700
   Human GTTGTTGAAGAATCACCATTATGTA----GAGCTACTAGGAATGTCTGAGGATGCAAGAGAGGAGTATATTTACCAGTTTTTTGAAGATAAGAGGTGGGC
     Dog TTTGTTGAAGAACCAGCATTCTGTA----GAGCTGCTGGGTATGTCCAAGGATGCAAGAAAAGAATACATTTACCAGTTCTTTGAAGACAAGAAGAGGGC
Pangolin TTTGTTGAAGAATCCGCATTCTGTATATAGAGCTACTGGGTGTGTCTAAGGATGCAAGAGAGGAGTATACTTACCAGTTTTTTGAAGAAAAGAAGTGGGC

         701                                                                                              800
   Human CATGAAAGTATTCAGTTCACTAAAAAGCAATGAGATGCTGTTTAGCATGTGCCAAGTCCCCCTAGTGTGCTGGGCCGCTTGTACTTGTCTGAAGCAGCAA
     Dog CTCGCAAGTATTCAGTTCACTAAGAAGCAATGAGATGCTTTTTAGCATGTGTAAAGTCCCCCTGGTATGCTGGGCCATCTGTACCTGTCTGGAGCAGCAA
Pangolin CAG--AAGTATTCAGTACACTGAAAAGTAATGAGATTCTTTTTGCCATGCATGAAGTCCCCATAGTGTGCTGGTC-ATTTGTCCTTGTCTGGAGCAACAA

         801                                                                                              900
   Human ATGGAGAAGGGTGGTGATGTCACATTGACCTGCCAAACAACCACAGCTCTGTTTACCTGCTATATTTCTAGCTTGTTCACACCAGTAGATGGAGGCTCTC
     Dog ATAGAAATGGGTGGTGATGTTTCATTAACCTGCAAAACAACAACAGCTCTGTTCACCCGCTATATTTCTAGCCTGTTCCCACCAGTAGATGGAAACTGTC
Pangolin ATGGAGAAGGATGGTGATCTCACATTAACCTGCAAAGCCACCAAAGCTCTGTTTACCTGCTGTATCTCTAGCTTGCTCACACCAGAAGATGGAAACT--C

         901                                                                                             1000
   Human CTAGTCTACCCAACCAAGCCCAGCTGAGAAGACTGTGCCAAGTCGCTGCCAAAGGAATATGGACTATGACTTACGTGTTTTACAGAGAAAATCTCAGAAG
     Dog CTAGTCTGCCCAATCAAACCCAACTAAAGAGCCTGTGCCACTTGGCTGCCAAAGGAGTGTGGACTATGACATCTGTGTTTTATAGGGAAGATCTCAGAAA
Pangolin CTAGTTTACCCAGTCAAGTC-AACTGAGGAGCCTGTGCCACTTGGCTGCCAAAGGAGTTTAGACTATGACAAGTGTTTTACAGGGA-AAAATCTCAGAAA

         1001                                                                                            1100
   Human GCTTGGGTTAACTCAATCTGATGTCTCTAGTTTTATGGACAGCAATATTATTCAGAAGGACGCAGAGTATGAAAACTGCTATGTGTTCACCCACCTTCAT
     Dog GCATGGCTTAACTAAATCAGATGTCTCGATTTTCCTGGACATGAATATTCTTCAAAAGGACACAGAGTATGAAAATTGCTATGTGTTCACTCACCTGCAT
Pangolin GCATGGGTTAACTAAATCTGATGTCTCAGTTTCCCTGGACATGAGTATTCTTCAGCAGGATGTGCAGTATGAGAACTGCAATGTGTTTACCCACCTCCAC

         1101                                                                                            1200
   Human GTTCAGGAGTTTTTTGCAGCTATGTTCTATATGTTGAAAGGCAGTTGGGAAGCTGGGAACCCTTCCTGCCAGCCTTTTGAAGATTTGAAGTCATTACTTC
     Dog GTTCAGGAGTTTCTTGCAGCTATGTTCTATATGTTAAGAGACAATTGGGAGACCAGGAATAATTTATTTGATTCTTTTGAAGATTTGAAGCTATTGCTTG
Pangolin ATTCAGGAGTTCTTTGCAGCTGTGTTCTATATGTTGAAAGGCAGTTGGGGAACCAAGGACCATTCCTTTCAGTCTTTACAAGGCTTGAAGCTGTTACTTG

         1201                                                                                            1300
   Human AAAGCACAAGTT----ATAAAGACCCCCATTTGACACAGATGAAGTGCTTTTTG----------------------------------------------
     Dog AAAGCAAGAGTT----CTAAAGATCCCCATTTGATGCAGATGAAATGCTTTTTG----------------------------------------------
Pangolin AAAGCAACAGTTGGTTATAAAGATCGCCATGTGACACAGATGAAATGTTCTTTGGCCTTTCTTTTTTAATTAAGGTATCATTGATATACGCTCTTATGAA

         1301                                                                                            1400
   Human ----------------------------------------------------------------------------------------------------
     Dog ----------------------------------------------------------------------------------------------------
Pangolin GGTTTCACATGGGCAACATCGTGGTTACTACATTTACCCGTATTATCAATTCTGGCCCCCACCTCCAAGCCCACTGCAGTCACTGTTTGTCAGCTTAGTA

         1401                                                                                            1500
   Human ----------------------------------------------------------------------------------------------------
     Dog ----------------------------------------------------------------------------------------------------
Pangolin AGATGTTACAGAGTCACTACTTGTCTTTGTGCTATACTGCCTTCCCCATGCCCACTCTACACTATGTGTGCTGATCCTAATGCTCCTTAATCCCCTTCTC

         1501                                                                                            1600
   Human ----------------------------------------------------------------------------------------------------
     Dog ----------------------------------------------------------------------------------------------------
Pangolin CCTCCCTTCCCACCTGCCCTCCCCCTCTGTTTTTCCTTTGGTAACTGCTAGTCCCTTCTTGGAGTTTGTGAGTCTGCTGCTGTTTTGTTCCTTCAGTTTT

         1601                                                                                            1700
   Human ----------------------------------------------------------------------------------------------------
     Dog ----------------------------------------------------------------------------------------------------
Pangolin TTCTTTGTTCTTATACTCCACATATGAGTGAAATCATTTGATACTTCTCTTTCTCTACCTGGCTTATTTCACTGAACATAATACCCTCTAGCTCCATCCA
```

```
         1701                                                                                              1800
   Human ----------------------------------------------------------------------------------------------------
     Dog ----------------------------------------------------------------------------------------------------
Pangolin TGTTGTTGCAAATGGTAGCATTTGTTTTCTTCCTATGGCTGAATAATATTCCATTGTGTATATGTACCACATCTTCTTTATCCATTCATCTACTGATGGA

         1801                                                                                              1900
   Human ----------------------------------------------------------------------------------------------------
     Dog ----------------------------------------------------------------------------------------------------
Pangolin CACTTAGGTTGCTACCATATCTTGGCTATTGTAAATAGTGCTGTGATAAACATAGGGGCGTCTTTTTGAATCTGGGATCGTGTTTTCTTAGGGTAAATTC

         1901                                                                                              2000
   Human --------------------TTTGGCCTTTTGAATGAAGATCGAGTAAAACAACTGGAGAGGACTTTTAACTGTAAAATGTCACTGAAGATAAAATCAAA
     Dog --------------------TTTGGCCTTTTGAATGAAGATCTACTAAAACAACTGGAGACAACTTTGAAATGTAGACTGTCACTGGAGATAAAAGGGAA
Pangolin CTAGGAGTGGAATTACTGTGTTTGGCCTTTTGAATGAAGATCAAGTAAAACAACTGGAAA-AACTTTTAATGATAAAATGCCACTGGATATAAAATGGAA

         2001                                                                                              2100
   Human GTTACTTCAGTGTATGGAAGTATTAGGAAACAGTGACTATTCTCCATCACAGCTGGGATTTCTGGAGTTGTTTCACTGTCTGTATGAGACTCAAGATAAA
     Dog AATCCTTCAGTGGCTGGAGATACTGGGAAACATCAAATGTTTTCCAGCAGAGCTAGAATTTCTGGAATTGTTTTTATGTCTGTATGAGACTCAAGATGAA
Pangolin ----CATCAGTGGATGGAACCATTAGGAAAC--TGACTATTTTTCATCACAGCTGGGATTTCTGGAGTTGTTCCACTGTCCACATGAGACTCAAGATTAC

         2101                                                                                              2200
   Human GCGTTTATAAGCCAGGCAATGAGATGTTTCCCAAAGGTTGCCATTAATATTTGTGAGAAAATACATTTGCTTGTATCTTCTTTCTGCCTTAAGCACTGCC
     Dog GCATTTATAAGCCAGGCAATGAGATCTTTCCAAAAGGTGGTCATTGATGTGTGTGGGAAAGTCCATTTGCTTGTGTCTTCATTCTGCCTTAAGCACTGCC
Pangolin ACATGTATAAGTCAGGCAATGAGATATTTCCAAACGGTTGCTGTTAATATTCGTG-GAAACTCCATTTGCTTGTGTCTTCATTCTGCCTTAAGCACTGCC

         2201                                                                                      2292
   Human GGTGTTTGCGGACCATCAGGCTGTCTGTAACTGTGGTATTTGAGAAGAAGATATTAAAAACAAGCCTCCCAACTAACACTTGGTAAGTGTGT
     Dog AATGTTTACAGACCATTAAATTATCTGTGACTGTGGTATTTGAGAAGACG---TTAAACTCAAGTCCCCCAGCTGAAATGTGGTAAGTGGGG
Pangolin AGGATTTATGGACCATAAAA----CTGTAACTGTGATATCTGAGAAGATGACCTCACACTCT------CCAGCTGAAGCATGGTTAAGTGTG
```

Supplementary Figure S2. ***NLRP14*** **is a pseudogene in the Malayan pangolin.** The nucleotide sequences of exon 3 of *NLRP14* of human, dog and Malayan pangolin were aligned. Nucleotides conserved in all three species are indicated by blue fonts. A pangolin-specific insertion is shown with red fonts. Deletions and insertions that lead to reading frame shifts are highlighted by red shading. The first in-frame stop codon is highlighted by magenta shading. Splicing signals at the borders of the flanking introns are shown with green shading. Nucleotide sequence accession numbers (GenBank): Human (NC_000011.10, nucl. 7042378-7043994), dog (NC_051825.1, nucl. 31537243-31538856), Malayan pangolin (NW_023436178.1, nucl. 449071-451326).
